# Mitogenomic and Ecological Perspectives on the Wide‐Ridged Indo‐Pacific Finless Porpoise *Neophocaena phocaenoides*: Toward Emerging Conservation Strategies

**DOI:** 10.1002/ece3.74249

**Published:** 2026-09-18

**Authors:** Manokaran Kamalakannan, Imon Abedin, Angkasa Putra, Vishwanath D. Hegde, Dhriti Banerjee, Jung Hwa Choi, Hyun‐Woo Kim, Shantanu Kundu

**Affiliations:** ^1^ Mammal and Osteology Section Zoological Survey of India Kolkata India; ^2^ Department of Zoology Bodoland University Kokrajhar India; ^3^ Interdisciplinary Program of Marine and Fisheries Sciences and Convergent Technology Pukyong National University Busan Republic of Korea; ^4^ Zoological Survey of India Western Ghat Regional Centre Kozhikode India; ^5^ Zoological Survey of India Prani Vigyan Bhawan Kolkata India; ^6^ Ocean and Fisheries Development International Cooperation Institute, College of Fisheries Science Pukyong National University Busan Republic of Korea; ^7^ Department of Marine Biology Pukyong National University Busan Republic of Korea; ^8^ Research Center for Marine Integrated Bionics Technology Pukyong National University Busan Republic of Korea; ^9^ Marine Integrated Biomedical Technology Center, National Key Research Institutes in Universities Pukyong National University Busan Republic of Korea; ^10^ Department of Biology, Faculty of Science and Technology Airlangga University Surabaya Republic of Indonesia; ^11^ International Graduate Program of Fisheries Science Pukyong National University Busan Republic of Korea

## Abstract

Prior to this study, the narrow‐ridged finless porpoise ( *Neophocaena asiaeorientalis* ) had been extensively studied across East Asia, whereas the wide‐ridged finless porpoise ( *Neophocaena phocaenoides* ) remained comparatively poorly characterized throughout its broad geographic range, particularly in the Indian Ocean. This study addresses this gap through morphological, mitogenomic, and ecological analyses of an *N. phocaenoides* carcass from Kozhikode Beach, Arabian Sea, India. The specimen was identified as *N. phocaenoides* based on the absence of a dorsal fin, a broad dorsal ridge, a blunt beakless head, spade‐shaped teeth, and a short broad rostrum. The comparative analysis of *Neophocaena* revealed conserved vertebrate gene organization, A + T‐rich composition, diverse codon usage, strong purifying selection across all protein‐coding genes, and nucleotide variation in the control region. The overall mean interspecific genetic distance among *Neophocaena* species and subspecies was 0.005. The Bayesian phylogenetic analysis and haplotype network reconstruction revealed high haplotype diversity (Hd = 0.9957) and consistently clustered the newly generated mitogenome with the previously reported Arabian Sea sequence, forming a distinct lineage separated from the Pacific populations. Additionally, multiple species delimitation analyses recovered six Operational Taxonomic Units across geographic regions, although broader sampling is required to validate these patterns across the Indian Ocean. Furthermore, ensemble Species Distribution Modeling identified 378,540 km^2^ of suitable habitat, primarily driven by bathymetry (61.92%). While significant adequate habitats were identified in China, Indonesia, and the Arabian Sea, only 23.37% of these areas overlap with existing Marine Protected Areas and Important Marine Mammal Areas. Moreover, high anthropogenic pressure was detected in high‐suitability regions, particularly in the South China Sea and Persian/Arabian Gulf. These findings underscore the vulnerability of *N. phocaenoides* and support the need for adaptive, regionally coordinated conservation strategies aligned with Convention on Biological Diversity and IUCN‐Cetacean Specialist Group frameworks.

## Graphical abstract

IUCN‐defined extant distribution of the Indo‐Pacific finless porpoise ( *Neophocaena phocaenoides* ), overlaid with occurrence records, global Marine Protected Areas (MPAs), and Important Marine Mammal Areas (IMMAs).

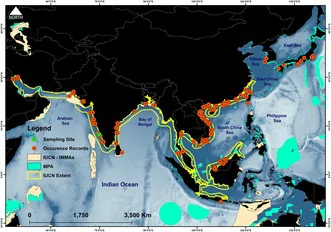

## 1 Introduction

Cetaceans, a group of aquatic mammals comprising whales, dolphins, and porpoises, include approximately 98 extant species that occupy diverse aquatic habitats ranging from oceans to freshwater rivers (Wilson and Mittermeier 2014). Among them, porpoises (family Phocoenidae) are closely related to the true dolphins (family Delphinidae), yet the two groups exhibit distinct morphological, anatomical, and behavioral traits (Jefferson 2002). Previous studies have suggested that porpoises originated in the temperate North Pacific Ocean during the Miocene epoch and subsequently dispersed into the Southern Hemisphere via the Indian and Atlantic Oceans (Murakami et al. 2012; Ben Chehida et al. 2020). All porpoises are small‐bodied, characterized by a blunt snout, absence of a beak, and spade‐shaped teeth. The family Phocoenidae comprises three genera, viz., *Neophocaena*, *Phocoena*, and *Phocoenoides*, and seven widely recognized extant species worldwide (Wilson and Mittermeier 2014).

The finless porpoise was first described by *G. Cuvier* in 1829 as *Delphinus phocaenoides* and was later reassigned to the genus *Neophocaena*, which for a long time was considered monotypic with three different geographic populations across the Indo‐Pacific Ocean (Reeves et al. 2003). However, subsequent assessments using various molecular markers, including haplotype analyses and morphological traits such as dorsal ridge structure and the extent of the tubercled area, revised their systematics (Gao and Zhou 1995; Wang et al. 2008; Jefferson and Wang 2011). Initially, two forms were recognized, viz., the wide‐ridged *Neophocaena phocaenoides* and the narrow‐ridged *Neophocaena asiaeorientalis* , which inhabits the coastal waters of the Indo‐Pacific Ocean (Collins et al. 2005). One population of the narrow‐ridged finless porpoise is believed to have dispersed into the Yangtze River in China, thereby establishing the only freshwater porpoise population in the world (Zhou et al. 2018). Subsequent population genomics research further identified two subspecies of *N. asiaeorientalis* , *N. asiaeorientalis asiaeorientalis* endemic to the mainstream Yangtze River and its associated river‐connected lakes, and *N. asiaeorientalis sunameri* distributed across the Yellow/Bohai Sea, the northern East China Sea, and the Japan Sea (Zhou et al. 2018).

These two megafaunal species face severe population declines driven primarily by habitat loss, compounded by anthropogenic pressures and the overexploitation of aquatic resources (Braulik et al. 2023). In response to these escalating threats, the IUCN has underscored the urgent need for comprehensive scientific assessments of these cetaceans and the development of targeted conservation strategies (Wang and Reeves 2017). Although recent research and conservation prioritization have largely focused on the two narrow‐ridged subspecies, the studies undertaken employ a multidimensional approach that integrates genetic, environmental DNA, and ecological investigations (Xia et al. 2005; Zheng et al. 2005; Li et al. 2011; Cheng et al. 2017; Mei et al. 2017; Lee et al. 2019; Ben Chehida et al. 2020; Liu et al. 2022; Lin, Karczmarski, et al. 2023; Lin, Zhao, et al. 2023; Qiao et al. 2024; Zhang et al. 2024; Kim et al. 2025; Lee et al. 2025; Rao et al. 2025). However, the Indo‐Pacific Finless Porpoise ( *N. phocaenoides* ), listed as “Vulnerable,” has received comparatively limited attention (Yoshida et al. 2001; Jayasankar et al. 2008; Koh et al. 2013; Patel et al. 2025). Recent genetic studies suggest that current population assessments may not fully capture the genetic distinctiveness of the finless porpoise species complex, particularly among populations along the west Indian Ocean, due to limited and geographically uneven sampling efforts (Jia et al. 2014; Lin et al. 2014; Lin et al. 2017; Lin, Karczmarski, et al. 2023; Lin, Zhao, et al. 2023). Although earlier studies on *N. phocaenoides* employed mitochondrial markers (the control region and cytochrome b) to infer population genetic structure, a comprehensive genomic assessment, particularly incorporating populations from the Indian Ocean remains lacking (Lin et al. 2017). Moreover, despite recent emergence in genetic research, the existing genomic data of *Neophocaena* species are heavily skewed toward populations inhabiting East Asian waters. Most available mitochondrial genomes of *Neophocaena* species are originated from the northwestern Pacific populations, especially along the Chinese coastline and adjacent seas (Liu et al. 2016; Cheng et al. 2017; Morin et al. 2023; Kim et al. 2025). Furthermore, although mitogenomic data have been widely used to interpret phylogeny and genetic diversity, the structural patterns and intra‐genomic variation within mitochondrial genomes have not been critically examined previously.

In addition, *N. phocaenoides* faces multiple severe threats, including habitat degradation, pollution, direct killings, and vessel collisions (Yang et al. 1999; Jefferson et al. 2002; Wang and Reeves 2017; Nithyanandan and Bohadi 2021). Specifically, the extensive coastal development, such as shrimp aquaculture, causeways, ports, and other shoreline infrastructure, has resulted in substantial loss and degradation of finless porpoise habitat throughout South and Southeast Asia, including the Persian/Arabian Gulf (Braulik et al. 2010). Moreover, pollution from industrial, agricultural, and domestic sources further intensifies environmental stress, contributing to deteriorating water quality and bioaccumulation of harmful contaminants (Braulik et al. 2010). Additionally, vessel collisions pose a serious and increasing risk to *N. phocaenoides* , particularly in regions with dense high‐speed ferry traffic (Parsons and Jefferson 2000). Together, these interacting threats have placed the wide‐ridged finless porpoise at considerable risk, highlighting the pressing need for focused research, enhanced monitoring, and strengthened conservation measures across its range. Additionally, the IUCN Cetacean Specialist Group (CSG) emphasizes the urgent need to identify suitable habitats for *N. phocaenoides* across the broader Indo‐Pacific region (Wang and Reeves 2017). Even with its wide distribution and threatened status, a comprehensive assessment of habitat suitability remains lacking. In this context, marine species distribution modeling (SDM) offers a robust framework for addressing this knowledge gap. While SDM has been extensively used in terrestrial systems, its application in marine environments is rapidly expanding and guiding conservation planning for marine megafauna (Marshall et al. 2014; Klaassen et al. 2025). Beyond identifying optimal habitat envelopes, these delineated areas can also be assessed for specific threats to the species, enabling a clearer understanding of risk factors within the habitat. This implementation is therefore essential for informing regional management strategies and prioritizing areas requiring immediate conservation action.

Hence, this study aims to (i) examine the external morphology and craniometric characteristics of *N. phocaenoides* from the Indian Ocean; (ii) reassess and analyze mitogenomic data of finless porpoises to evaluate genomic characteristics, haplotype diversity, phylogenetic relationship, and operational taxonomic units (OTUs) identification; (iii) identify the extent of suitable habitat for *N. phocaenoides* across the Indo‐Pacific region using an ensemble SDM framework; and (iv) assess habitat suitability within Marine Protected Areas (MPAs) and Important Marine Mammal Areas (IMMAs), and evaluate anthropogenic pressures within country‐wise maritime boundaries within the identified suitable habitats. The application will support the development of evidence‐based management and conservation strategies for *N. phocaenoides* in alignment with the Convention on Biological Diversity (CBD) and IUCN‐CSG frameworks.

## 2 Materials and Methods

### 2.1 Sampling and Species Identification

**FIGURE 1:**
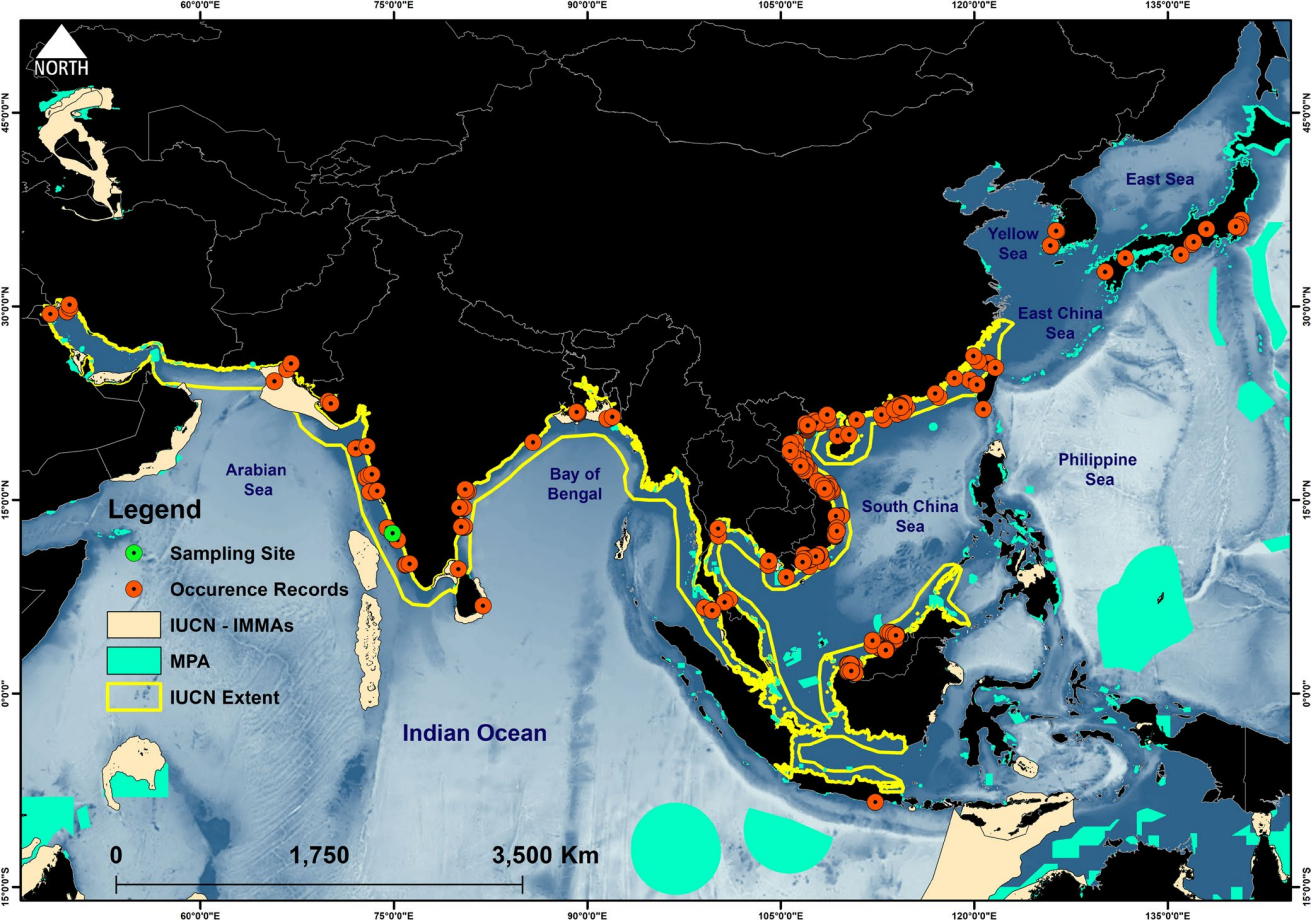
IUCN‐defined extant range of the Indo‐Pacific finless porpoise ( *N. phocaenoides* ), overlaid with occurrence records derived from the present sampling sites and secondary data sources. The spatial data on global Marine Protected Areas (MPAs) were obtained from the Protected Planet database (https://www.protectedplanet.net/en, accessed on 20 November 2025), while Important Marine Mammal Areas (IMMAs) were provided by the IUCN Marine Mammal Protected Areas Task Force obtained through personal communication.

A single carcass of finless porpoise drifted in and was found stranded on Kozhikode beach (11.287501°N, 75.758696°E) on the Arabian Sea coast of Kerala, Indian Ocean (Figure 1). A tissue sample was collected from the specimen to avoid surface contamination and preserved in 70% ethanol at the Western Ghat Regional Centre (WGRC), Zoological Survey of India (ZSI), Kozhikode, Kerala and was subsequently used for molecular analyses. No specific permits or ethical approvals were required for this study, as the sample was obtained from a naturally deceased animal. The species was identified as *N. phocaenoides* based on the available taxonomic keys from an earlier study including the absence of a dorsal fin, a broad dorsal ridge, a blunt beakless head, spade‐shaped teeth, and a short broad rostrum (Jefferson et al. 2015). The carcass was buried in alluvial soil mixed with crystal salt by excavating a pit approximately 2 ft deep, placing the carcass inside, and fully covering it to allow natural decomposition while maintaining skeletal articulation. After 2 months of burial, the specimen was exhumed and the articulated skeleton was recovered with minimal disturbance to its anatomical arrangement. The bones were rinsed thoroughly with clean water to remove adhering soil and decomposed tissue remnants. The final cleaning and whitening were performed by immersing the bones in a 10% hydrogen peroxide solution for approximately 30 min, which effectively removed residual organic matter and improved the visibility of morphological features. The processed skeletal material (Voucher No. ZSIWGRC_3626) was subsequently dried, labeled, and deposited as an articulated reference specimen at the National Zoological Collections of the WGRC, ZSI where it is currently housed for scientific study and future reference.

### 2.2 Ethics Statement

All experimental procedures were conducted in India in compliance with the regulations of the host institutions and the provisions of the Biological Diversity Act, 2002, the Biological Diversity Rules, 2004, and the Biological Diversity (Amendment) Act, 2023, and all procedures adhered to the ARRIVE 2.0 guidelines for animal research (Percie du Sert et al. 2020). The generated sequence data have been deposited in a publicly accessible global database and are freely available for worldwide use, in accordance with the recommendations adopted at the 16th Conference of the Parties (COP16) to the CBD, held in November 2024, and endorsed by the Parties to the CBD (196 countries) (Rouard et al. 2025).

### 2.3 DNA Extraction and Mitogenome Sequencing

The molecular experiments, including mitogenome sequencing with assembly, were performed at Unipath Specialty Laboratory Ltd (https://www.unipath.in/), Ahmedabad, India. The mitochondrial DNA was isolated using the Alexgen DNA Kit (Alexius Biosciences, Gujarat, India) (Ahmad et al. 2007). The DNA quantification was carried out utilizing a Qubit 4.0 fluorometer to ensure accuracy. The paired‐end sequencing library was prepared using the QIAseq FX DNA Library Kit (CAT‐180479). The DNA was fragmented into smaller segments using the Covaris M220 Focused Ultrasonicator (Covaris Inc., San Diego, CA, USA), followed by adapter ligation at both ends of the fragments for Illumina sequencing compatibility. In addition, to maximize yield from limited input material, the HiFi PCR Master Mix (Takara Bio Inc., Kusatsu, Shiga, Japan) was used for high‐fidelity amplification. Moreover, the prepared libraries were analyzed on the TapeStation 4150 (Agilent Technologies, Santa Clara, CA, USA) using High Sensitivity D1000 ScreenTape, following the standard protocol. After determining the DNA concentration and peak size from the TapeStation results, the library was sequenced on the Illumina NovaSeq 6000 platform (Illumina, San Diego, CA, USA) for cluster generation and sequencing. The resulting high‐quality paired‐end reads (~20 million) were assembled and annotated using Geneious Prime v2023.0.1 (Kearse et al. 2012). The gene boundaries and strand orientations were confirmed using the MITOS Galaxy web server (https://mitos.bioinf.uni‐leipzig.de) and MitoAnnotator (https://mitofish.aori.u‐tokyo.ac.jp/annotation/input/) (Bernt et al. 2013; Iwasaki et al. 2013). The Protein‐coding genes (PCGs) were validated by ensuring the sequences of amino acid conformed to the vertebrate mitochondrial genetic code using the ORF Finder tool (https://www.ncbi.nlm.nih.gov/orffinder/), while initiation and termination codons were determined based on the reference mitochondrial genome of the same species (accession numbers KC777291). The generated mitogenome was submitted to GenBank using the Sequin submission tool under the GenBank Accession No. OR353710, along with the gene feature file with accurately defined boundaries and strand orientations.

### 2.4 Mitogenome Characterization and Comparative Analyses

The circular mitogenome of *N. phocaenoides* was visualized and manually curated to detect the intergenic spacers and overlapping regions. The nucleotide composition and sequence lengths of PCGs, ribosomal RNAs (rRNAs), transfer RNAs (tRNAs), and control region (CR) were quantified using MEGA v12 (Kumar et al. 2024). To investigate the genetic diversity at both intra and inter‐species levels, an additional PCGs dataset (11,425 bp) was constructed with all available mitogenomes (generated + GenBank database, *n* = 89) of *Neophocaena* species. The genetic distances were calculated using the standard Kimura 2‐parameter (K2P) model through MEGA v12. The number of haplotypes, haplotype diversity (Hd), and nucleotide diversity (π) were estimated by using DnaSP v.6.0 (Rozas et al. 2017). The haplotype networks were constructed using the Templeton, Crandall, and Sing (TCS) method embedded in POPART (Clement et al. 2000; Leigh and Bryant 2015). The codon saturation in the PCGs was evaluated in DAMBE v6 by examining the relative rates of transitions and transversions between *Neophocaena* species (Xia 2017). Additional analyses were conducted to determine the relative synonymous codon usage (RSCU), amino acid composition, as well as pairwise comparisons of synonymous (Ks) and nonsynonymous (Ka) substitution rates between *Neophocaena* species and sub‐species using DnaSP v6.0. The CR was characterized to delineate its structural domains based on established literature, and comparative analyses were performed (Sbisà et al. 1997).

### 2.5 Mitogenome‐Based Phylogenetic Inferences and Species‐Delimitation

To clarify the matrilineal phylogenetic relationship of *Neophocaena* species and subspecies level, 88 complete mitochondrial genomes were retrieved from the GenBank database. The mitogenomes of *Phocoena phocoena* (Accession no. AJ554063) and *Phocoenoides dalli* (Accession no. MT948081) were included as outgroup taxa for the phylogenetic analysis. For phylogenetic construction, all 13 PCGs were concatenated and aligned using iTaxoTools v0.1 (Vences et al. 2021). The optimal nucleotide substitution model was determined with jmodeltest v2, which selected GTR + G + I as the best fit (Darriba et al. 2012). The Bayesian Inference (BI) was applied to construct a well‐supported phylogenetic framework. The BI topology was constructed using MrBayes v3.1.2 with nst = 6, one cold chain, and three hot Metropolis‐coupled Markov chain Monte Carlo (MCMC) chains (Ronquist et al. 2012). This analysis ran for 10,000,000 generations with tree sampling every 100 generations, discarding 25% of samples as burn‐in. The convergence was considered achieved when the average standard deviation of split frequencies reached ≤ 0.01 and the Potential Scale Reduction Factor (PSRF) values approached 1 for all estimated parameters. The BI tree was visualized and refined using iTOL v7 web server (https://itol.embl.de/login.cgi) (Letunic and Bork 2024). The distance‐based species delimitation methods Automatic Barcode Gap Discovery (ABGD) and Assemble Species by Automatic Partitioning (ASAP) were applied to identify OTUs among all *Neophocaena* mitogenomes (Puillandre et al. 2012; Puillandre et al. 2021). The aligned FASTA file was used as input for both analyses, which were performed under the Kimura substitution model using iTaxoTools v0.1.

### 2.6 Study Range and Occurrence Data

The Indo‐Pacific finless porpoise occurs in a narrow band of shallow coastal marine waters along the northern rim of the Indian and western Pacific Oceans, ranging from the Persian/Arabian Gulf eastwards around the Indian Ocean to the Indo‐Malay region, extending further to Java, Indonesia, and northwards to the Taiwan Strait and central Chinese waters (Wang and Reeves 2017). Given the limited dedicated survey effort across much of its suspected range, the current IUCN distribution maps also include areas of possible or projected habitat, where oceanographic conditions align with the species known ecological preferences (Wang and Reeves 2017). Accordingly, this work adopted an extended range from the Persian/Arabian Gulf to the East Sea, consistent with the IUCN distribution and incorporating recently reported confirmed sightings (Ku and Choi 2022; Kim et al. 2025; Patel et al. 2025). The species presence records were compiled from secondary sources reporting verified sightings, bycaught specimens, or literature‐based occurrences. A total of 404 occurrence points post the year 2000 were obtained from the IUCN Geospatial Conservation Assessment Tool (GeoCAT), which aggregates records from multiple reputable repositories (Bachman et al. 2011). Further, to reduce spatial autocorrelation among occurrence points, spatial rarefaction was performed at a resolution of 31.36 km^2^ using the spatial rarefaction function in SDM Toolbox v2.4 (Brown et al. 2017). This rarefaction scale matched the raster resolution used in modeling, helping to minimize redundancy and reduce the risk of model overfitting. After filtering, 103 unique occurrences were retained for the final habitat suitability modeling. Notably, pseudo‐absence points were generated across the entire study extent, with their number set equal to the number of presence points (*n* = 103) to ensure balanced representation.

### 2.7 Variables for Marine Distribution Model

The environmental predictor variables were obtained from the Bio‐ORACLE database v3.0, which provides 26 physical, chemical, biological, and topographic marine data layers at a global scale on a uniform grid with a spatial resolution of 0.05° (~5.6 km) (Assis et al. 2024). The present‐day (2010–2020) surface‐layer conditions were downloaded, as these variables are most relevant to the ecology of the focal species. All raster layers were standardized to a common spatial resolution and clipped to match the extent of the study area. Further, to address spatial multicollinearity among predictors, the study employed the SAHM (Software for Assisted Habitat Modeling) package implemented in VisTrails (Morisette et al. 2013). The variables showing strong pairwise correlations (Pearson, Spearman, or Kendall coefficients *r* > 0.8) were excluded to minimize redundancy and improve model interpretability (Warren et al. 2010). This filtering procedure reduced collinearity while retaining key environmental gradients (Figure S1). The final covariate set consisted of 10 variables after accounting for inter‐variable correlations.

### 2.8 Ensemble Distribution Model

The habitat suitability modeling was conducted using an ensemble framework that integrated five algorithms: Boosted Regression Trees (BRT), Multivariate Adaptive Regression Splines (MARS), Generalized Linear Models (GLM), Maximum Entropy (MaxEnt), and Random Forests (RF) (Guisan et al. 2007; Elith and Leathwick 2009; Miller 2010). This ensemble approach leveraged the complementary strengths of individual algorithms to better capture the ecological complexity underlying species distributions, thereby enhancing predictive accuracy and robustness (Hao et al. 2020). All models were implemented in the SAHM package within VisTrails (Talbert and Talbert 2012; Morisette et al. 2013). The model outputs were generated as continuous probability maps ranging from 0 (least suitable) to 1 (most suitable). These surfaces were converted to binary presence–absence predictions using the sensitivity = specificity (SES) threshold criterion, with models retained if they exceeded an AUC threshold of 0.75 (Lavazza et al. 2023). An ensemble agreement map was then constructed with values ranging from 0 to 5, indicating the number of algorithms predicting a given pixel as suitable habitat. A value of 5 denoted complete consensus across all algorithms, allowing detailed assessment of spatial agreement and habitat configuration. The model performance was evaluated using multiple metrics, including area under the receiver operating characteristic curve (AUC), true skill statistic (TSS), Cohen's Kappa, proportion correctly classified (PCC), sensitivity, and specificity for both training datasets and 10‐fold spatial block cross‐validation replicates (Cohen 1968; Allouche et al. 2006; Phillips and Elith 2010; Jiménez‐Valverde et al. 2013).

### 2.9 Protected Areas and Threat Assessment

The MPAs for West Asia and the Asia–Pacific region were obtained from the Protected Planet database (https://www.protectedplanet.net/en, assessed on 20 November 2025), the authoritative global source of spatial data on protected areas and other effective area‐based conservation measures (OECMs) (UNEP‐WCMC and IUCN 2025). In addition, the IMMAs, discrete ocean regions identified as critical habitats for marine mammal species, were incorporated into the analysis. Although IMMAs are not legally designated PAs, they provide robust, science‐based guidance for policymakers and conservation planners. The IMMAs shapefiles used in this study were requested from the IUCN Marine Mammal Protected Areas Task Force IMMAs knowledge product and received via personal communication (IUCN Marine Mammal Protected Areas Task Force 2024). Furthermore, to assess anthropogenic pressures within the habitat of *N. phocaenoides* , a cumulative human impact layer was employed (Halpern et al. 2012). This layer integrates multiple threat categories, including land‐based activities, climate‐related impacts, fisheries, and shipping, to generate a comprehensive cumulative anthropogenic impact layer. The data were obtained from the Ocean Health Index website at a spatial resolution of 10 km (https://oceanhealthindex.org/resources/data/, assessed on 20 November 2025). This source provides a reputable and thorough assessment of threats within the habitat of *N. phocaenoides* conducted within national maritime boundaries within the range (https://www.marineregions.org/downloads.php).

## 3 Results

### 3.1 External and Skeleton Morphology

**FIGURE 2:**
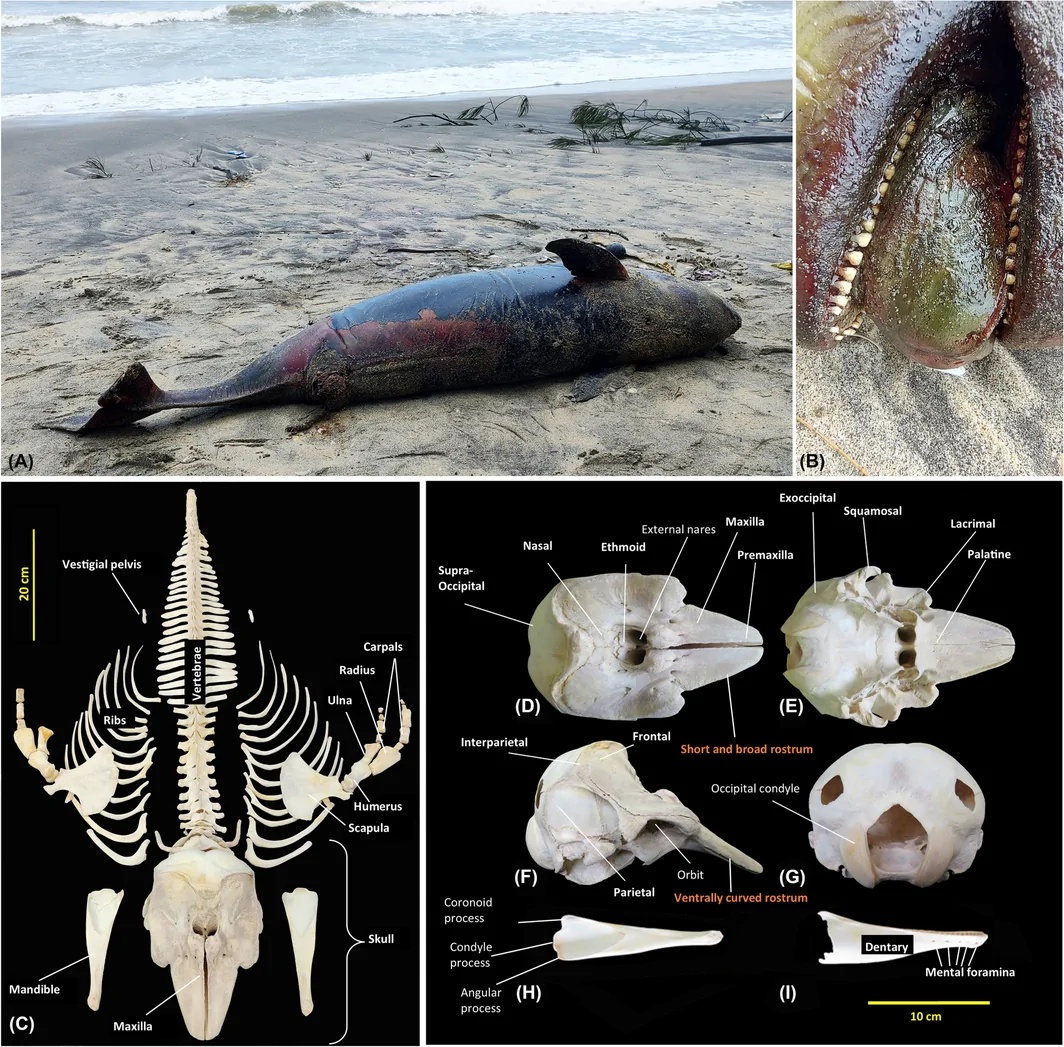
(A) The carcass of *N. phocaenoides* collected from Kozhikode Beach, Kerala, India, (B) spade‐shaped teeth as a distinguished character, (C) articulated skeleton and (D–I) views of the cranium: (D) dorsal, (E) ventral, (F) lateral, and (G) posterior views. (H–I) Views of the dentary: (H) lateral and (I) medial of *N. phocaenoides* deposited in the National Zoological Collections of WGRC, ZSI (Voucher No. ZSIWGRC_3626). All photographs were taken by the first author (Manokaran Kamalakannan) by using Sony RX10 IV and manually edited by Adobe Photoshop CS 8.0.

The collected specimen was characterized by a large, blunt melon‐shaped head without a distinct beak and a shallow depression posterior to the blowhole (Figure 2A). The body coloration was generally black to dark gray, with comparatively lighter patches around the lips and throat. The specimen lacked a dorsal fin and instead possessed a broad, low midline dorsal ridge beginning posterior to the mid‐body region. The flippers were long and tapering, and the tail fluke was deeply notched with a concave trailing edge. The measured head‐to‐tail length was 144 cm, tail width 40 cm, and flipper length 24 cm.

The teeth in both the upper and lower jaws were distinctly flattened laterally and spade‐shaped (Figure 2B). The articulated skeleton includes the complete vertebral column with associated ribs, paired scapulae, humeri, radii, ulnae, and distal carpal elements, reflecting the typical forelimb morphology of small odontocetes (Figure 2C). The skull possessed a short and broad rostrum with a broad premaxillary plate (Figure 2D,E), and the rostrum was distinctly deflected ventrally (Figure 2F). The occipital condyles were well defined posteriorly (Figure 2G). The condylobasal length of the skull measured 185 mm, while the rostrum length was 65 mm. The mandible was complete, with the dentary showing distinct coronoid, condylar, and angular processes (Figure 2H). Multiple mental foramina were present along the anterior portion of the dentary (Figure 2I).

### 3.2 Mitogenomic Characterization

**FIGURE 3:**
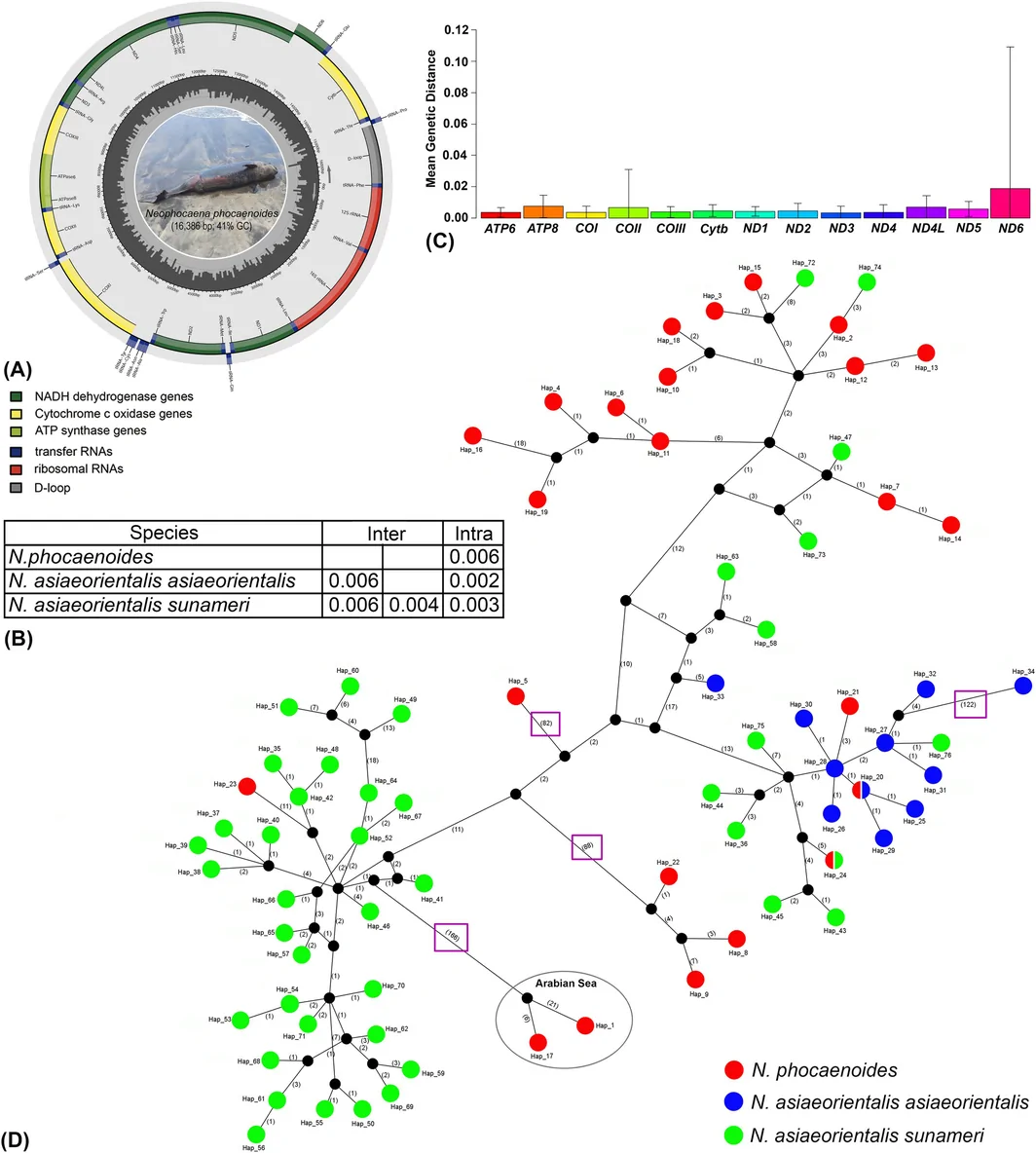
(A) Circular representation of the complete mitochondrial genome of *N. phocaenoides* , visualized using the MitoAnnotator online web server (https://mitofish.aori.u‐tokyo.ac.jp/annotation/input/). Genes encoded on the heavy and light strands are shown on the inner and outer circles of the map, respectively. PCGs, rRNAs, tRNAs, and the non‐coding CR are distinguished by different colors, indicating their strand orientation and genomic boundaries. The numbers indicate the genomic positions from the start to the end of the mitogenome. A photograph of a representative individual was taken by the first author (Manokaran Kamalakannan) and manually merged using Adobe Photoshop CS 8.0. (B) Mean inter‐ and intra‐specific genetic distances among *Neophocaena* species/subspecies estimated using 13 concatenated mitochondrial PCGs. (C) Comparative mean genetic distances for each of the 13 mitochondrial PCGs across *Neophocaena* species. (D) Inter‐species and sub‐species haplotype network of *Neophocaena* based on 13 mitochondrial PCGs, generated using the Templeton–Crandall–Sing (TCS) method implemented in POPART. The purple boxes with numbers exhibiting higher numbers of segregating sites among the different *Neophocaena* haplotypes, whereas the black circle highlights the unique haplotype identified in the Arabian Sea.

The complete mitochondrial genome of *N. phocaenoides* was 16,386 bp in length and exhibited the typical vertebrate gene organization, comprising 37 genes (13 PCGs, two rRNA genes, 22 tRNA genes) and one CR (Figure 3A). Most of the genes were transcribed from the heavy (H) strand, whereas *ND6* and eight tRNA genes were encoded on the light (L) strand. The rRNA genes, *12S rRNA* (973 bp) and 16S rRNA (1580 bp), were positioned between *tRNA‐Phe* and *tRNA‐Leu* (L2). The lengths of the tRNA genes ranged from 61 bp for *tRNA‐Ser* (S1) to 75 bp for *tRNA‐Leu* (L2). Among the PCGs, *ND5* was the longest (1824 bp), while *ATP8* was the shortest (201 bp). Most PCGs initiated translation with the ATG start codon, although *ND2* and *ND5* used ATT and ATA, respectively. The identified stop codons included TAA, AGA, and incomplete forms (TA‐ or T–) (Table 1). However, in *N. asiaeorientalis sunameri* (Accession No. KT852939) from East China Sea, canonical TAA and TAG stop codons are present in seven PCGs, whereas four genes (*ND4*, *ND2*, *ND3*, and *COIII*) harbor truncated termination codons (T– or TA‐), with *COI* terminating in AGG and *Cytb* in AGA. In contrast, the Yangtze River subspecies, *N. asiaeorientalis asiaeorientalis* (Accession No. KP170488) displays a slightly different pattern with ten of the thirteen PCGs initiate with ATG, while *ND2* uses ATT as well as *ND3* and *ND5* use ATA. Five genes (*COII*, *ATP6*, *ND4L*, *ND5*, and *ND6*) terminate with TAA, *Cytb* ends with AGA, *COI* with AGG, *ND1* and *ATP8* with TAG, and four genes (*ND2*, *COIII*, *ND3*, *ND4*) possess truncated stop codons (T– or TA‐). In addition, the intergenic spacers varied in size, with the longest (32 bp) located between *tRNA‐Asn* and *tRNA‐Cys*, while the gene overlaps occurred in several regions, most notably between *ATP8* and *ATP6* (40 bp) (Table 1).

**TABLE 1:** Structural organization of the complete mitochondrial genome of *N. phocaenoides* , presenting the genomic positions, strand orientation, gene lengths, and intergenic nucleotide.

Gene	Start	End	Strand	Size (bp)	Intergenic nucleotide	Anti‐codon	Start codon	Stop codon
*tRNA‐Phe (F)*	1	70	H	70	0	GAA		
*12S rRNA*	71	1043	H	973	–1			
*tRNA‐Val (V)*	1043	1109	H	67	0	TAC		
*16S rRNA*	1110	2689	H	1580	0			
*tRNA‐Leu (L2)*	2690	2764	H	75	1	TAA		
*ND1*	2766	3727	H	962	0		ATG	TA—
*tRNA‐Ile (I)*	3728	3796	H	69	–3	GAT		
*tRNA‐Gln (Q)*	3794	3866	L	73	3	TTG		
*tRNA‐Met (M)*	3870	3938	H	69	0	CAT		
*ND2*	3939	4980	H	1042	0		ATT	T– —
*tRNA‐Trp (W)*	4981	5049	H	69	5	TCA		
*tRNA‐Ala (A)*	5055	5123	L	69	1	TGC		
*tRNA‐Asn (N)*	5125	5198	L	74	32	GTT		
*tRNA‐Cys (C)*	5231	5297	L	67	0	GCA		
*tRNA‐Tyr (Y)*	5298	5364	L	67	1	GTA		
*COI*	5366	6911	H	1546	0		ATG	T– —
*tRNA‐Ser (S2)*	6912	6980	L	69	7	TGA		
*tRNA‐Asp (D)*	6988	7055	H	68	0	GTC		
*COII*	7056	7739	H	684	3		ATG	TAA
*tRNA‐Lys (K)*	7743	7810	H	68	1	TTT		
*ATP8*	7812	8012	H	201	−40		ATG	TAA
*ATP6*	7973	8653	H	681	−1		ATG	TAA
*COIII*	8653	9437	H	785	0		ATG	TA—
*tRNA‐Gly (G)*	9438	9506	H	69	0	TCC		
*ND3*	9507	9853	H	347	0		ATA	TA—
*tRNA‐Arg (R)*	9854	9923	H	70	0	TCG		
*ND4L*	9924	10,220	H	297	−7		ATG	TAA
*ND4*	10,214	11,591	H	1378	0		ATG	T– —
*tRNA‐His (H)*	11,592	11,659	H	68	0	GTG		
*tRNA‐Ser (S1)*	11,660	11,720	H	61	1	GCT		
*tRNA‐Leu (L1)*	11,722	11,791	H	70	0	TAG		
*ND5*	11,792	13,612	H	1824	−17		ATA	TAA
*ND6*	13,596	14,123	L	528	0		ATG	TAA
*tRNA‐Glu (E)*	14,124	14,192	L	69	4	TTC		
*Cytb*	14,197	15,336	H	1140	0		ATG	AGA
*tRNA‐Thr (T)*	15,337	15,406	H	70	−1	TGT		
*tRNA‐Pro (P)*	15,406	15,473	L	68	0	TGG		
Control Region	15,474	16,386		913				

*Note:* The symbols “H” and “L” indicate coding on the heavy and light strands, respectively, whereas “–” represents an incomplete stop codon.

### 3.3 Genetic Distance and Inter‐Taxa Haplotype Diversity

The overall mean K2P genetic distance across the PCG sequences of *Neophocaena* species was 0.005 (Tables S1, S2). Both *N. phocaenoides* and *N. asiaeorientalis* exhibited a genetic distance of 0.006 between them, whereas the two recognized subspecies of *N. asiaeorientalis* showed a comparatively lower divergence of 0.004. In contrast to *N. asiaeorientalis* , *N. phocaenoides* displayed a higher level of intra‐specific genetic variation (0.006) (Figure 3B). Among the analyzed PCGs, *ND6* and *ATP8* exhibited the highest mean interspecific genetic distances (0.019 ± 0.090 and 0.008 ± 0.007, respectively) compared with the other 11 PCGs (Figure 3C). The inter‐taxa haplotype network analysis further demonstrated a mixed haplotypic distribution among all three nominal taxa, comprising 76 haplotypes, 648 segregating sites, Hd = 0.9957, and π = 0.0851. A single haplotype of *N. asiaeorientalis asiaeorientalis* (Hap_34: KP170488) and several other haplotypes of *N. phocaenoides* (Hap_5: BK071568; Hap_8: BK071562; Hap_9: BK071561; Hap_22: MT948057) exhibited substantial divergence from their nearest median vectors representing unsampled haplotypes. However, the lack of associated metadata for these *Neophocaena* mitogenomes in public databases precludes the reliable assignment of highly divergent populations to their designated taxonomic identities in GenBank and their respective geographic origins. The newly generated mitogenome (Hap_17; Accession OR353710) in the present study clustered closely with Hap_1 (Accession PX427675), both originating from the Arabian Sea, and shared the highest number of segregating sites (*n* = 166) relative to their median vector and other haplotypes (Figure 3D). Collectively, the observed haplotype diversity patterns suggest that the *N. phocaenoides* population in the Arabian Sea may exhibit genetic differentiation from other regional populations in the Indo‐Pacific Ocean. However, given the limited sampling from the Arabian Sea, these patterns may require further validation through broader geographic coverage and more comprehensive population‐level sampling.

### 3.4 Nucleotide Composition, Codon Usage, and Substitutions Pattern

**FIGURE 4:**
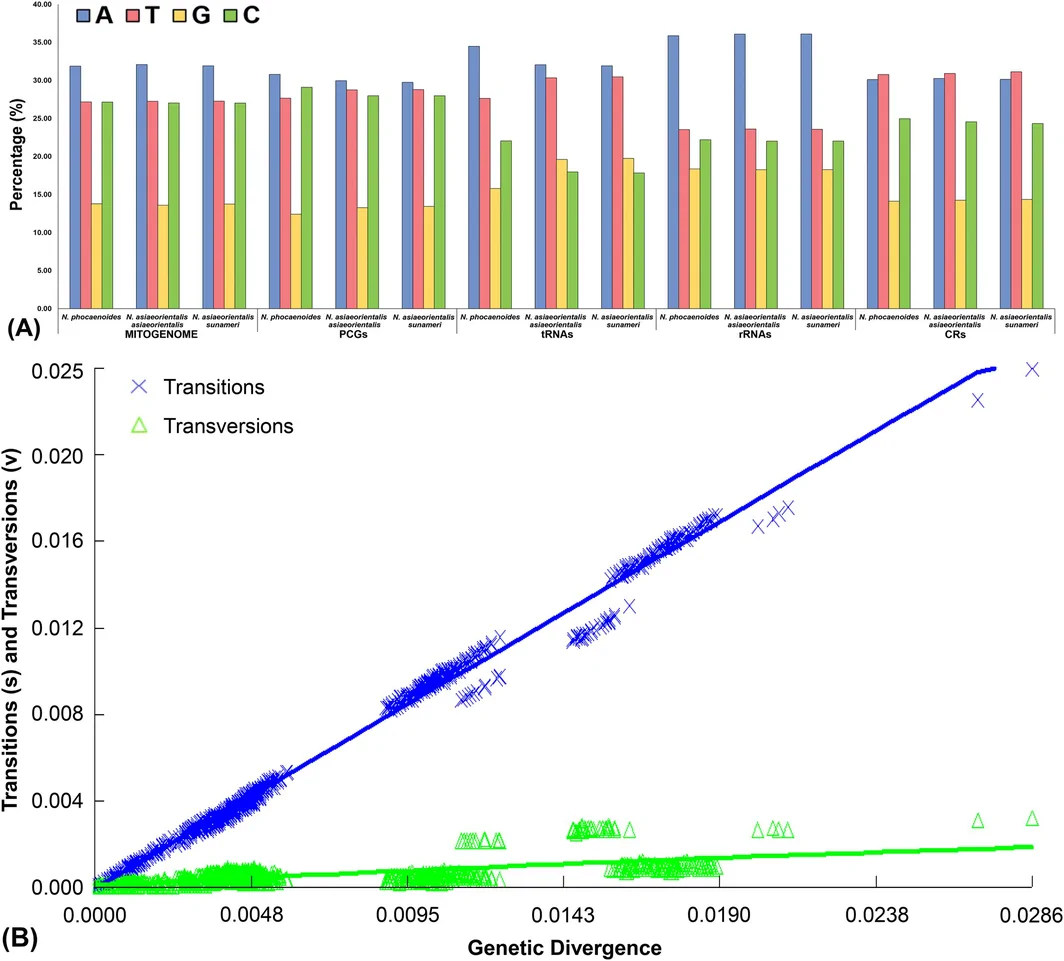
(A) The bar chart visualizing the nucleotide composition percentages across the complete mitogenome of three *Neophocaena* species and subspecies. (B) Scatter plot showing the relationship between transitions (s) and transversions (v) with genetic divergence in PCGs, based on substitution distances.

**FIGURE 5:**
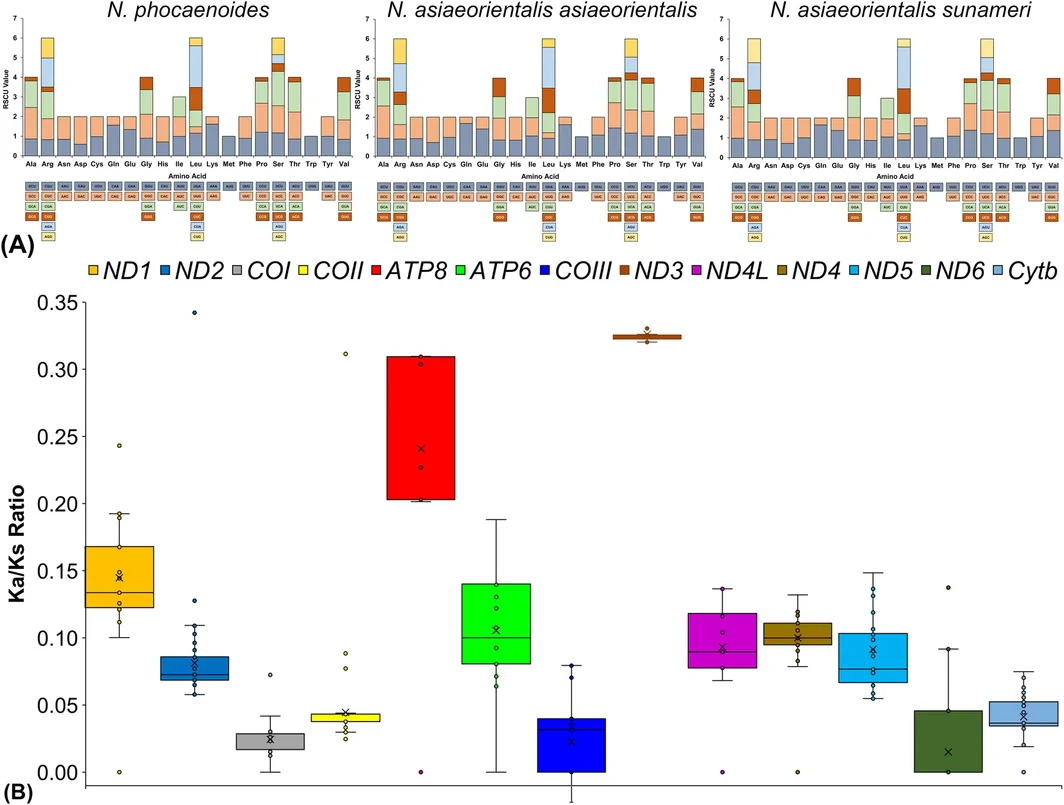
(A) The comparative analysis of relative synonymous codon usage (RSCU) among three *Neophocaena* species/subspecies. The collective RSCU values are plotted on the *y*‐axis against codons corresponding to each amino acid on the *x*‐axis. (B) The boxplot illustrating the pairwise Ka/Ks ratios for each mitochondrial PCG among *Neophocaena* taxa.

The comparative analysis of nucleotide composition showed that the mitogenomes of *N. phocaenoides* , *N. asiaeorientalis asiaeorientalis* , and *N. asiaeorientalis sunameri* are dominated by A + T bases. Notably, variation among species was more prominent in the PCGs, with *N. phocaenoides* exhibiting higher proportions of adenine and cytosine, whereas *N. asiaeorientalis sunameri* showed greater abundances of thymine and guanine (Figure 4A). The analysis of saturation highlighted that neither transitions nor transversions had reached saturation, as genetic divergence values consistently increased across all PCGs among the analyzed *Neophocaena* mitogenomes (Figure 4B). The amino acid compositions of *N. phocaenoides* , *N. asiaeorientalis asiaeorientalis* , and *N. asiaeorientalis sunameri* presented noticeable variation, with leucine exhibiting the most abundant amino acid (2.13%, 2.08%, and 2.11%, respectively), while alanine displayed the lowest abundance (0.19%, 0.12%, and 0.16%, respectively). The RSCU analysis further demonstrated that codons encoding arginine, leucine, and serine were used most frequently, with up to six synonymous codons represented, whereas methionine and tryptophan were the least variable, each being encoded by a single codon (Figure 5A; Table S3). All 13 PCGs reflected pairwise Ka/Ks values below “1,” consistent with strong purifying selection among all *Neophocaena* species. The pairwise average Ka/Ks ratios spanned from 0.015 ± 0.027 for *ND6* to 0.326 ± 0.013 for *ND3*, following the pattern: *ND6* < *COIII* < *COI < Cytb < COII* < *ND2* < *ND5* < *ND4L* < *ND4* < *ATP6* < *ND1* < *ATP8* < *ND3* (Figure 5B; Table S4).

### 3.5 Control Regions Characteristics

The multiple sequence alignment of the CR across *Neophocaena* species, including newly generated sequences and sequences retrieved from GenBank (*n* = 89), elucidated nucleotide variations distributed throughout the Extended Termination‐Associated Sequences (ETAS) domains, encompassing ETAS‐1 and ETAS‐2, as well as within the Conserved Sequence Blocks (CSB) domains, comprising CSB‐1, CSB‐2, and CSB‐3. The lengths of these segments were 64 bp for ETAS‐1, 59 bp for ETAS‐2, as well as 24 bp, 17 bp, and 18 bp for CSB‐1, CSB‐2, and CSB‐3, respectively (Figures S2–S4). Despite the observed sequence variation, ETAS‐1 and ETAS‐2 contained 61 and 56 conserved positions, respectively, with thymine being the predominant nucleotide, accounting for 26 positions in ETAS‐1 and 23 positions in ETAS‐2. The CSB‐1 comprised 18 conserved positions, with adenine being the most frequent nucleotide, occurring at nine positions. In contrast, the fully conserved motifs within CSB‐2 and CSB‐3 were predominantly characterized by repetitive cytosine residues.

### 3.6 Phylogenetic Relationships and Operational Taxonomic Units

**FIGURE 6:**
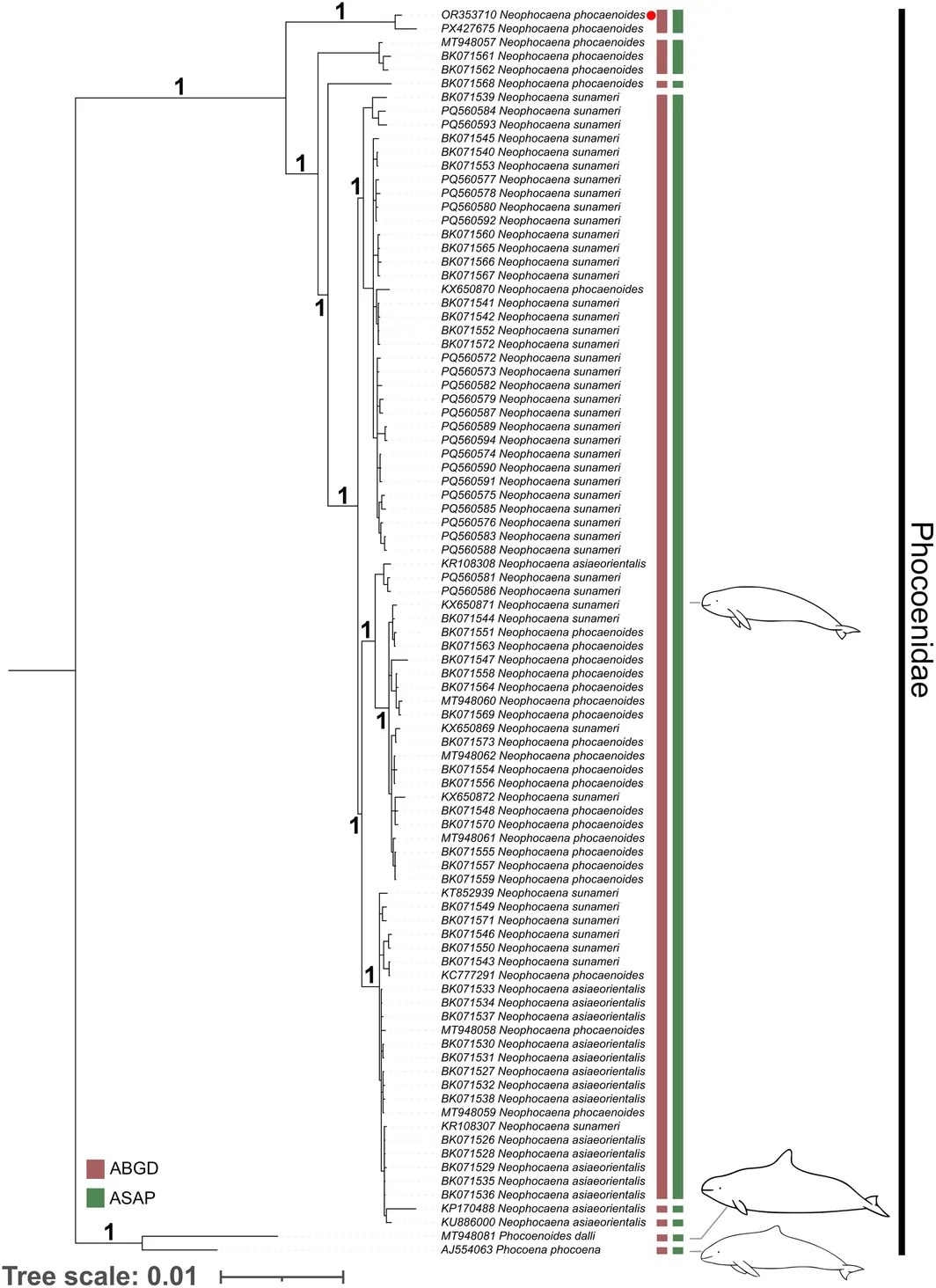
Bayesian phylogenetic tree inferred from concatenated sequences of 13 mitochondrial PCGs clearly distinguishing *Neophocaena* taxa from other cetacean species and revealing a polytomies within the genus. Posterior probability support values are shown as black numerals at the corresponding nodes. The newly generated mitogenome is indicated by a red dot. The Operational taxonomic unit (OTU) delimitations inferred by ABGD and ASAP analyses are indicated by distinct dark maroon and dark green side bars, respectively. Representative illustrations of the three major groups within the family Phocoenidae were obtained from the open‐access repository Wikimedia Commons and manually assembled using Adobe Photoshop CS8.0.

The BI phylogenetic analysis based on the concatenated 13 PCGs from complete mitogenomes showed monophyletic clustering of *Neophocaena* species and sub‐species with high posterior probability support (Figure 6). Several deeper nodes within *Neophocaena* received high support, with posterior probability values of 1.0, indicating strong support for major internal groupings. Nevertheless, the phylogeny did not recover the three nominal *Neophocaena* taxa as completely reciprocally monophyletic lineages. Instead, substantial intermixing of sequences assigned to *N. phocaenoides* , *N. asiaeorientalis asiaeorientalis* , and *N. asiaeorientalis sunameri* was observed across the tree. Moreover, several apparent soft polytomies were present within the *Neophocaena* topology. These were characterized by multiple terminal lineages arising from very closely spaced internal nodes and by extremely short branch lengths. The ABGD and ASAP species‐delimitation approaches consistently identified six OTUs across the analyzed *Neophocaena* mitogenomes. The newly generated mitogenome (Accession OR353710) grouped with a previously reported Arabian Sea mitogenome (Accession PX427675), and both were assigned to a single OTU in ABGD and ASAP analyses (Figure 6; Table S5).

### 3.7 Ensemble Model Evaluation

The ensemble model incorporating the five algorithms exceeded the AUC threshold, demonstrating strong predictive performance. The training AUC values ranged from 0.885 to 0.955, while cross‐validation AUC values ranged from 0.862 to 0.895, thus surpassing the threshold in both phases (Table 2; Figure S5). Among the individual models, BRT achieved the highest AUC in both training and cross‐validation, whereas RF yielded the lowest in training and MARS the lowest in cross‐validation. The difference between training and cross‐validation performance (ΔAUC) was greatest for MARS and lowest for RF, with BRT and GLM exhibiting identical ΔAUC values. Beyond AUC, additional evaluation metrics (TSS, Kappa, PCC, sensitivity, and specificity) also indicated consistently strong performance across both training and cross‐validation datasets for all models included in the ensemble.

**TABLE 2:** Model evaluation assessment for each algorithm.

Model	Dataset	AUC	ΔAUC	PCC	TSS	Kappa	Specificity	Sensitivity
BRT	Train	0.955	0.060	86.300	0.723	0.710	0.866	0.857
	CV	0.895		81.600	0.623	0.609	0.823	0.800
GLM	Train	0.929	0.060	83.700	0.674	0.657	0.835	0.839
	CV	0.869		76.400	0.530	0.511	0.763	0.767
MARS	Train	0.928	0.066	88.200	0.769	0.752	0.876	0.893
	CV	0.862		83.000	0.638	0.631	0.854	0.783
MAXENT	Train	0.93	0.038	82.900	0.661	0.641	0.825	0.836
	CV	0.892		79.000	0.586	0.564	0.782	0.803
RF	Train	0.885	0.008	79.700	0.590	0.575	0.804	0.786
	CV	0.893		81.800	0.599	0.598	0.866	0.733

Abbreviations: AUC, Area under the curve; BRT, Boosted regression tree; GLM, Generalized linear model; MARS, Multivariate adaptive regression splines; MaxEnt, Maximum entropy; PCC, Proportion correctly classified; RF, Random forest; TSS, True skill statistic.

### 3.8 Variables Importance and Responses

The model evaluation indicated that the selected environmental variables contributed unevenly to the prediction of species distribution. The topographic variable bathymetry emerged as the dominant predictor, with a mean contribution of 61.92%, underscoring the strong influence of depth on habitat suitability (Table 3). The chemical variable iron (11.80%) and the topographic variable slope (11.09%) were also important determinants. Furthermore, the biological productivity variable represented by chlorophyll (6.64%) and the topographic variable aspect (6.17%) provided an additional contribution to the model. In contrast, temperature, silicate, salinity, and current velocity made only minor contributions, suggesting that these variables play a limited role in shaping the present distribution of the species.

**TABLE 3:** Predictor importance of each algorithm within the ensemble framework and mean variable importance for habitat suitability of *N. phocaenoides* .

Variable	BRT	GLM	MARS	MAXENT	RF	Mean	Mean (%)
Aspect	0.000	0.017	0.049	0.016	0.001	0.017	6.167
Bathymetry	0.257	0.236	0.136	0.195	0.015	0.168	61.917
Chlorophyll	0.000	0.000	0.045	0.042	0.003	0.018	6.642
Curr_Velocity	0.000	0.000	0.000	0.004	0.000	0.001	0.311
Iron	0.070	0.045	0.021	0.023	0.001	0.032	11.802
Phosphate	0.000	0.017	0.000	0.008	0.000	0.005	1.863
Salinity	0.000	0.000	0.000	0.001	0.000	0.000	0.071
Silicate	0.000	0.000	0.000	0.001	0.000	0.000	0.127
Slope	0.000	0.133	0.000	0.017	0.000	0.030	11.094
Temp	0.000	0.000	0.000	0.000	0.000	0.000	0.004

Variable responses indicated consistent patterns across modeling algorithms in the ensemble model for the species distribution. Bathymetry consistently emerged as the most influential predictor across all algorithms, with a steep increase in suitability in shallow waters, indicating a strong restriction of the species to coastal depths (Figure S6). Among the chemical variables, iron exhibited a positive relationship with suitability, with higher concentrations corresponding to greater occurrence probability. The topographic variables also contributed notably, as slope was associated with higher suitability in areas of moderate inclination, whereas aspect showed elevated suitability in particular orientations. The biological productivity variable, chlorophyll, displayed a unimodal response, with habitat suitability maximized at moderate concentrations. In contrast, temperature, salinity, silicate, phosphate, and current velocity exhibited relatively flat or weak response curves, indicating that these variables exert only minor influence compared to bathymetry, nutrient availability, and productivity. Collectively, the models suggest that the present distribution of the species is primarily constrained by bathymetric and nutrient‐related factors, while other environmental gradients act as secondary determinants.

### 3.9 Identification of Suitable Habitat

**FIGURE 7:**
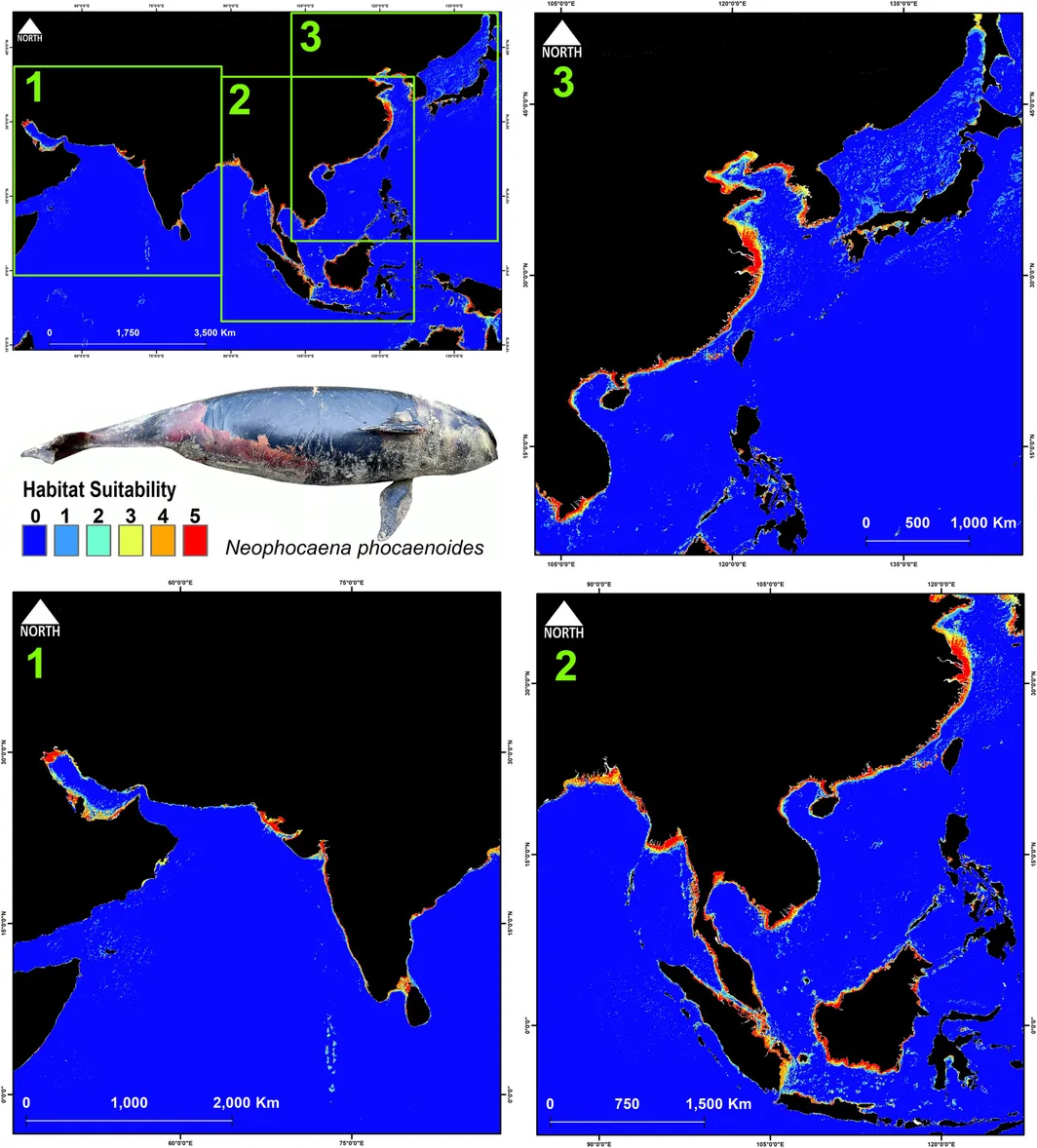
Maps showing the predicted suitable habitat extent for *N. phocaenoides* based on ensemble species distribution model across the entire study area, with zoomed‐in panels (1–3). Habitat suitability is classified into five levels (1–5) representing increasing model agreement, where higher classes indicate greater consensus among individual models. Class “0” denotes unsuitable habitat with zero model agreement, whereas Class “5” indicates complete model agreement and thus suitable habitat. The species photograph was taken by the first author (Manokaran Kamalakannan).

**FIGURE 8:**
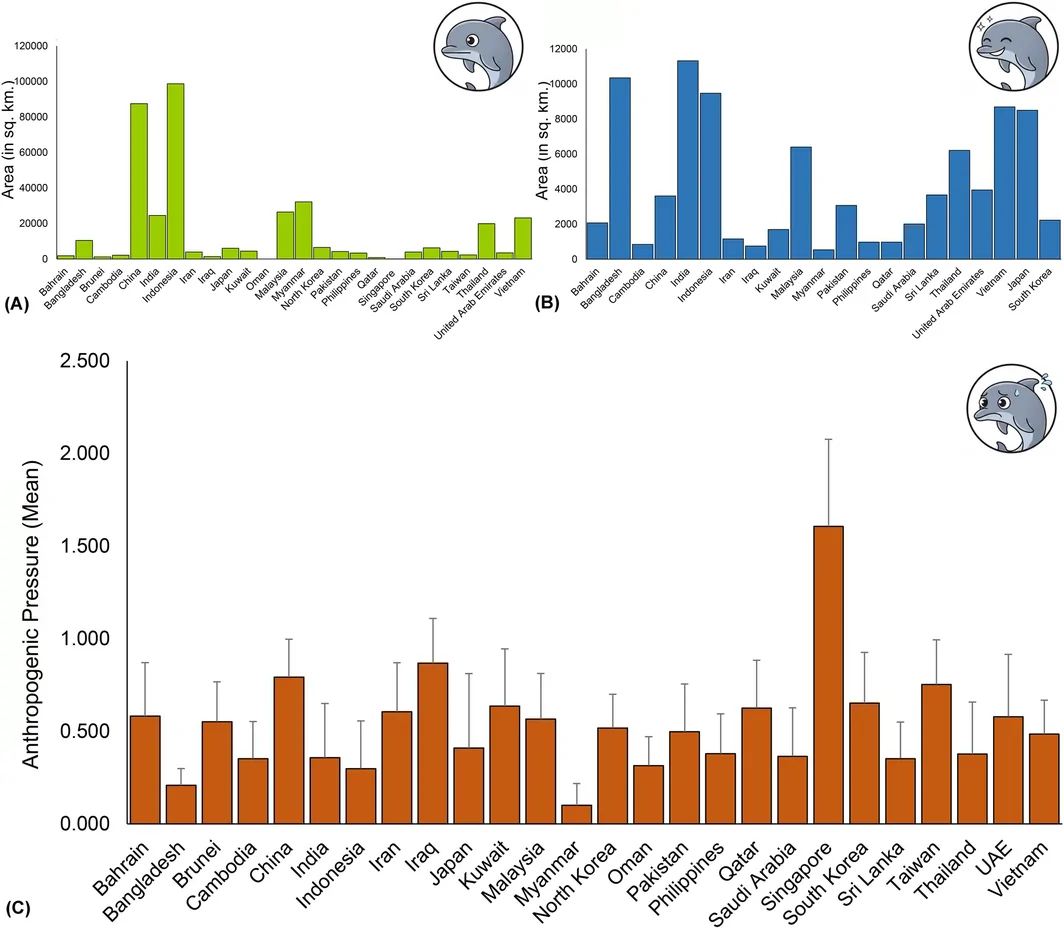
(A) Extent of suitable habitat (km^2^), (B) proportion of suitable habitat (km^2^) overlapping with Marine Protected Areas (MPAs) and Important Marine Mammal Areas (IMMAs), and (C) mean cumulative human impact (anthropogenic pressure) across the suitable habitat of *N. phocaenoides* within national maritime boundaries. Species icons were generated using the Google Gemini AI platform.

The ensemble model identified 378,540 km^2^ of suitable habitat within the training extent. However, within the IUCN‐assessed range (2,946,852 km^2^), only 256,742 km^2^ was predicted as suitable, representing just 8.71% of the total range (Figure 7). The spatial distribution of suitable habitats for *N. phocaenoides* shows marked regional variation across Asia. The highest suitable area was identified in Indonesia (98,666 km^2^) and China (87,482 km^2^), highlighting these regions as key strongholds for the species (Figure 8A). Additionally, moderate extents of optimal habitat occurred in Myanmar (32,163 km^2^), Malaysia (26,421 km^2^), India (24,545 km^2^), Vietnam (23,118 km^2^), Thailand (19,928 km^2^), Japan (6042 km^2^), South Korea (6268 km^2^) etc., indicating substantial potential along the Indo‐Pacific coastal belt. In contrast, Gulf countries such as Bahrain (1801 km^2^) and Saudi Arabia (3903 km^2^) exhibited limited habitat availability, while Oman showed no suitable areas.

### 3.10 Protected Area and Threat Assessment

The assessment of protected area (PA) coverage within maritime boundaries revealed that only 88,466 km^2^ of the total suitable habitat for the species falls within designated protection zones, representing merely 23.38% of the identified suitable extent (Figure 8B; Table S6). This coverage includes both MPAs and IMMAs. Among countries within the study extent, India and Bangladesh contained the largest proportions of protected optimal habitats, covering approximately 11,320 km^2^ and 10,348 km^2^, respectively, indicating relatively strong integration of porpoise habitats within national marine conservation frameworks. Furthermore, several countries in Southeast Asia and the Western Pacific, including Indonesia (9470 km^2^), Vietnam (8686 km^2^), Japan (8498 km^2^), and Malaysia (6397 km^2^) exhibited moderate protection levels, suggesting partial safeguarding of critical coastal and nearshore environments. In contrast, Middle Eastern nations such as Bahrain (2069 km^2^), Saudi Arabia (2007 km^2^) along with Asian countries such as Pakistan (3073 km^2^) demonstrated comparatively limited representation of suitable habitats within protected boundaries. Overall, these findings emphasize that although certain portions of the species' adequate range fall within protected areas, large expanses of high‐suitability habitats across South and Southeast Asia remain unprotected, underscoring the need for expanded spatial conservation planning and transboundary protection efforts.

**FIGURE 9:**
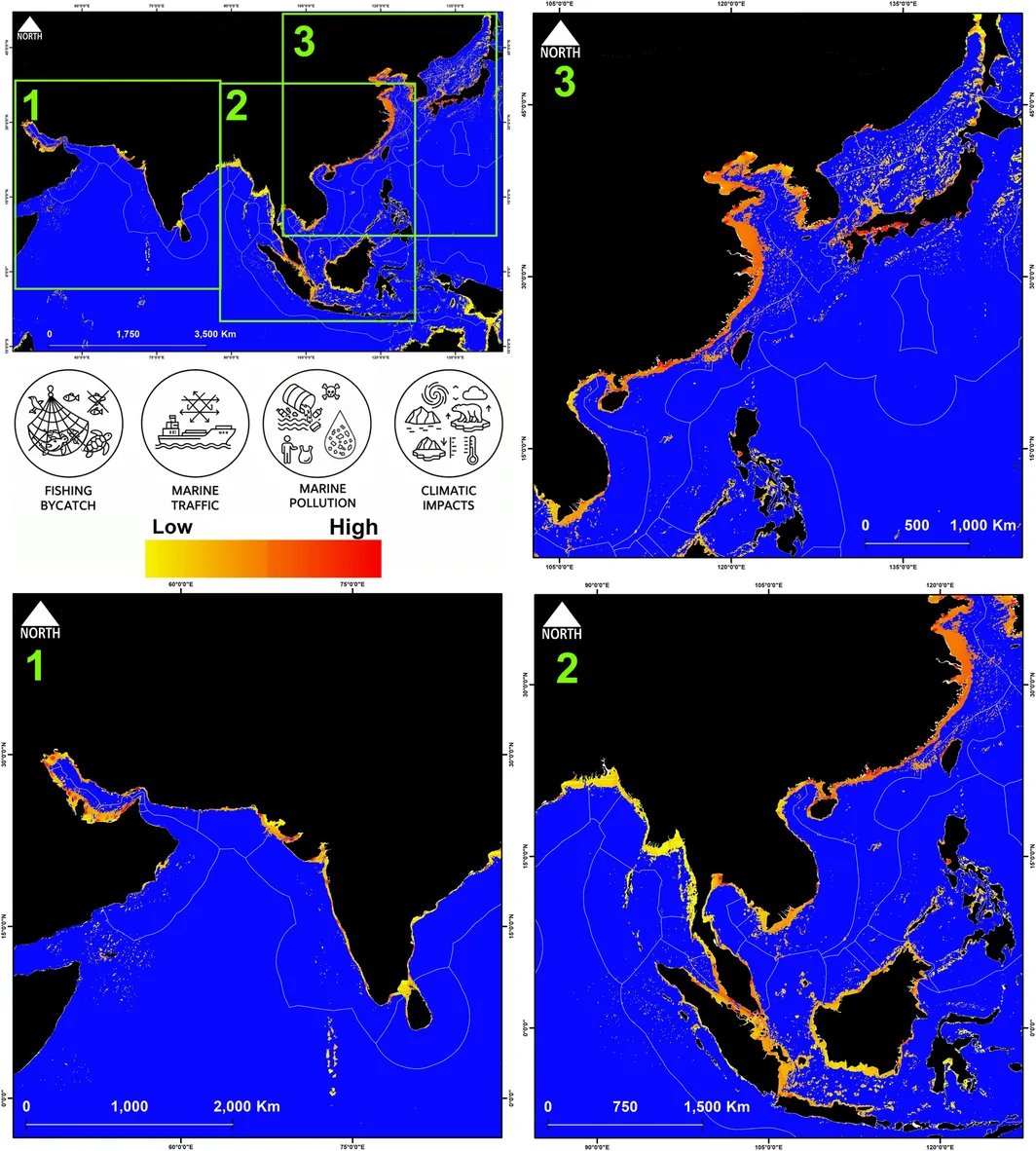
Maps showing the spatial overlap between cumulative human impact (anthropogenic pressures) and predicted suitable habitat for *N. phocaenoides* within the national maritime boundaries across the entire study area, with zoomed‐in panels (1–3). Anthropogenic pressure icons were generated using the Google Gemini AI platform to visually represent major threats categories.

In terms of anthropogenic pressure, the estimated mean pollution within the suitable habitat extent exhibited considerable spatial variability across the assessment range (Figure 8C; Table S7). The highest levels of pollution were concentrated in the South China Sea and adjacent regions, particularly around Singapore (1.607 ± 0.470), China (0.793 ± 0.204), and Taiwan (0.752 ± 0.242). Likewise, substantial anthropogenic pressure was also evident in the Persian/Arabian Gulf, where Iraq (0.868 ± 0.242), Kuwait (0.636 ± 0.310), Qatar (0.625 ± 0.258), and Iran (0.605 ± 0.265) experienced notable pollution overlaps within adequate habitat zones (Figure 9). Notably, moderate pressures were recorded in several South and Southeast Asian countries, including Brunei (0.552 ± 0.215), Pakistan (0.497 ± 0.258), Vietnam (0.409), and India (0.357 ± 0.293). In contrast, Indonesia (0.299 ± 0.257), Bangladesh (0.209 ± 0.090), and Myanmar (0.100 ± 0.118) were among the regions showing the lowest levels of marine disturbance and anthropogenic overlap within suitable habitats.

## 4 Discussion

The external morphology and skull characters observed in the present specimen are consistent with the diagnostic features previously described for *N. phocaenoides* (Jefferson and Wang 2011; Jefferson et al. 2015). The absence of a dorsal fin, presence of a broad dorsal ridge, short and broad ventrally deflected rostrum, and distinctly spade‐shaped teeth collectively distinguish *N. phocaenoides* from *N. asiaeorientalis* , which typically possesses a relatively longer and narrower rostrum and a comparatively elevated dorsal ridge (Jefferson and Wang 2011). The morphometric measurements of the specimen, including total body length (144 cm), condylobasal length (185 mm), and rostrum length (65 mm), fall within the known range reported for *N. phocaenoides* , in which the total body length ranges from 135 to 171 cm, condylobasal length from 181 to 245 mm, and rostrum length from 62 to 92 mm (Jefferson et al. 2015). In contrast, *N. asiaeorientalis* generally exhibits comparatively larger measurements, with total body length ranging from 130 to 227 cm, condylobasal length from 210 to 295 mm, and rostrum length from 77 to 97 mm (Jefferson and Wang 2011), further supporting the taxonomic identification of the stranded individual. The external colouration observed in the present specimen should be interpreted cautiously because colouration in stranded cetaceans may change rapidly following death due to post‐mortem decomposition processes (Wilson and Mittermeier 2014). Nevertheless, the general dark gray dorsal colouration with lighter ventral regions broadly agrees with previous descriptions of *N. phocaenoides* .

Over the past few decades, habitat degradation and escalating anthropogenic pressures have emerged as critical threats to the biodiversity of marine ecosystems worldwide (Selig et al. 2014; Braun et al. 2023). These pressures undermine the functional integrity of marine and coastal ecosystems, reducing their capacity to support associated species that depend on stable environmental and ecological conditions (Flensborg et al. 2023). Consequently, biodiversity loss in this environment is occurring at a faster and more severe rate than in terrestrial ecosystems (Knapp et al. 2017; Ramírez et al. 2017). To address these challenges, both genetic and ecological research are advancing rapidly worldwide, providing essential insights for safeguarding a wide range of marine fauna (Roberts et al. 2017; Manel et al. 2020; Fan et al. 2022). In this context, cetaceans represent a lineage of marine mammals that have undergone profound macroevolutionary transitions, resulting in remarkable physiological and morphological adaptations that enable them to thrive in highly challenging marine environments (McGowen et al. 2014). Several partial genes and mitogenomes of *Neophocaena* species have been sequenced across Indo‐Pacific region; however, most analyses have focused on genetic diversity and phylogenetic relationships, with comparatively limited emphasis on structural features and genomic variation. The newly generated *N. phocaenoides* mitogenome showed the conserved mitochondrial architecture typical of vertebrates. The comparative analysis revealed variation in PCG initiation and termination patterns among *Neophocaena* taxa, consistent with previous findings (Liu et al. 2016; Cheng et al. 2017). The ATG was the predominant start codon, supporting the conserved mitochondrial initiation pattern reported in vertebrates, whereas stop codons were more variable (Donath et al. 2019). The TAA stop codon was commonly observed, while the occurrence of AGA in *Neophocaena* species/subspecies indicates lineage‐associated variation in termination signals (Lind et al. 2013; Liu et al. 2019). The observed amino acid composition and codon usage patterns, including the higher representation of codons for arginine, leucine, and serine and lower representation of methionine and tryptophan, suggest conserved functional and translational constraints on mitochondrial PCGs (Shen et al. 2019; LaBella et al. 2019). Moreover, pairwise Ka/Ks ratios below “1” across PCGs indicate strong purifying selection, reflecting evolutionary constraints that maintain mitochondrial gene function (Shtolz and Mishmar 2019). The CR also showed characteristic nucleotide patterns within the ETAS and CSB domains, consistent with earlier reports (Sbisà et al. 1997). In particular, conserved T‐rich ETAS‐1 and A‐rich CSB‐1 regions may be associated with mitochondrial replication and transcription, whereas repetitive C residues in CSB‐2 and CSB‐3 may contribute to structural or regulatory functions of the CR (Fernández‐Silva et al. 2003; Jemt et al. 2015).

Furthermore, the taxonomic classification of marine mammals, including cetaceans, is inherently complex and frequently debated because it requires delineating distinct boundaries within a continuum of evolutionary divergence (Taylor, Archer, et al. 2017; Taylor, Perrin, et al. 2017). This complexity is further intensified by the limited diagnostic value of morphological characters, pronounced variation in body size, and the extensive ecological diversity of cetaceans such as *Neophocaena* species, collectively reinforcing the need for high‐resolution molecular approaches (Morin et al. 2023). Accordingly, the mitogenome‐based phylogenetic analysis conducted in this study confirms the monophyly of Phocoenidae, consistent with previous findings based on partial or genomic approaches (Slater et al. 2010; Ben Chehida et al. 2020; McGowen et al. 2020). Nevertheless, the low genetic divergence between *N. phocaenoides* and the two subspecies of *N. asiaeorientalis* , together with the observed phylogenetic patterns, indicate complex relationships among these taxa. This clustering pattern is further deepened by the presence of six OTUs and the occurrence of overlapping haplotype clusters. These findings align with previous reports from East Asian waters, also showing low genetic diversity in *N. asiaeorientalis* based on partial mitochondrial genes (Yoshida et al. 2001; Xia et al. 2005; Zheng et al. 2005; Jayasankar et al. 2008; Koh et al. 2013; Jia et al. 2014; Lee et al. 2019; Ku and Choi 2022; Patel et al. 2025). Moreover, the weaker resolution in the observed phylogenetic relationship among *Neophocaena* taxa is consistent with earlier analyses based on complete mitogenomes (Ben Chehida et al. 2020; Kim et al. 2025). The present findings corroborate earlier assertions that mitogenome sequences and their associated taxonomic identifications at species and sub‐species level available in the GenBank database require thorough verification, as they may reflect misidentifications or ambiguous collection records and warrants further validation.

Concurrently, the haplotype analysis of all available mitogenomes in the GenBank database corroborates the complex interrelationships among *Neophocaena* taxa. Notably, the *N. phocaenoides* cluster (dominated by red vertex) closely associated with few haplotypes of *N. asiaeorientalis sunameri* (Hap_47, Hap_72, Hap_73, and Hap_74), whereas the predominant cluster of *N. asiaeorientalis sunameri* similarly includes the haplotypes of *N. phocaenoides* (Hap_23). A similar pattern is also observed within the *N. asiaeorientalis asiaeorientalis* cluster (dominated by the blue vertex), which includes several unique and shared haplotypes of *N. phocaenoides* and *N. asiaeorientalis sunameri* . Nonetheless, several haplotypes also exhibit high numbers of segregating sites viz., Hap_34 ( *N. asiaeorientalis asiaeorientalis* ) with 122 sites; Hap_5, Hap_8, Hap_9, and Hap_22 ( *N. phocaenoides* ) with 82–88 sites as well as the Arabian Sea cluster (Hap_1 and Hap_17) of *N. phocaenoides* with 166 sites. The haplotype cluster identified from the Arabian Sea (Indian Ocean) in this study possibly reflects pronounced regional genetic structuring, consistent with earlier inferences based on partial mitochondrial loci (Lin et al. 2017). The possible distinct genetic structure of *N. phocaenoides* in the Indian Ocean is likely shaped by geographic isolation and regional oceanographic processes, as supported by the presence of major genetic barriers inferred using Monmonier's algorithm (Lin et al. 2017). However, confirmation of this pattern requires additional population‐level genetic data, particularly from the Bay of Bengal. Comparable population differentiation in cetacean species has been documented in the Arabian Sea, where colder surface waters, seasonal upwelling, and elevated primary productivity are driven by the southwest (summer) monsoon, whereas convective mixing and relatively warmer waters are associated with the northeast (winter) monsoon (Singh et al. 2011; Pomilla et al. 2014). Furthermore, evidence suggests that during the estimated period of cetacean population expansion from the southern Indian Ocean, the Arabian Sea experienced highly variable climatic conditions between approximately 60 and 18 kya, coinciding with the Last Glacial Age (Banakar et al. 2010; Ivashchenko and Clapham 2012). In line with this interpretation, the Arabian Sea (Indian Ocean) population of *N. phocaenoides* was consistently recovered as a single OTU in both ABGD and ASAP analyses, indicating that it possibly represents a distinct and evolutionarily significant conservation unit.

Notably, the ensemble model identified suitable habitats for this species along the Arabian Sea coast, extending eastward toward the East China Sea. The strong performance of the ensemble model can be attributed to the high thresholds achieved during training and cross‐validation, reflecting the robustness and quality of the environmental variables used. These layers effectively capture the macroecological preferences of the species and, when applied appropriately, enable the development of highly accurate species distribution models for marine taxa consistent with findings from previous studies (Tyberghein et al. 2012; do Amaral et al. 2015). Moreover, these variables have proven instrumental in understanding habitat relationships and environmental preferences of finless porpoise and other cetaceans, which are known to exhibit specific affinities for hydrological conditions (Braulik et al. 2012; Li, Lai, and Devlin 2021; Li, Lai, Liu, et al. 2021; Li, Lai, et al. 2022; Li, Deng, et al. 2022). The model identified bathymetry as the dominant predictor, contributing a mean of 61.92%, underscoring the critical influence of depth on habitat suitability. The chemical variable iron, an essential micronutrient that regulates phytoplankton growth and marine primary productivity, accounted for 11.80% of the contribution, while the topographic variable slope contributed 11.09%, indicating that both were important determinants (Tagliabue et al. 2017). Additionally, chlorophyll concentration (6.64%), representing biological productivity, and aspect (6.17%) provided meaningful contributions to the model. Collectively, these variables emphasize the significance of topographic and oceanographic features that universally shape the ecological niches of finless porpoises. The results reinforce previous findings that cetacean distributions are primarily governed by water depth, productivity, and other factors influencing prey abundance and availability (MacLeod 2009; Palacios et al. 2013). Such parameters often serve as reliable oceanographic proxies for prey distribution, further supporting the observed patterns (Goetsch et al. 2023).

The ensemble model estimated approximately 378,540 km^2^ of suitable habitat within the training extent. However, within the IUCN‐assessed range only 256,742 km^2^ was identified as suitable representing just 8.71% of the total range. This limited distribution is concerning, as it overlaps extensively with coastal regions subject to intense anthropogenic pressures. Prior research has documented threats from overfishing, industrial development, maritime transport, and human interference, all of which contribute to habitat degradation and population decline (Patel et al. 2025). Even more critical is that only 23.38% of the identified optimal areas fall within existing marine protected zones, highlighting a serious conservation gap that necessitates immediate management intervention. Furthermore, regions such as the Bay of Bengal and the South China Sea coincide with dense networks of commercial shipping lanes, where frequent vessel strikes and accidents have been reported (Wang and Reeves 2017). These vessels also contribute to increasing underwater noise pollution, which is another significant anthropogenic stressor for cetaceans. Studies have shown that exposure to high‐frequency vessel noise can disrupt porpoise foraging behavior, suppress echolocation activity, and reduce prey capture success (Wisniewska et al. 2018; Frankish et al. 2023).

### 4.1 Future Direction for Research and Conservation

Given these pressing concerns, it is imperative to strengthen spatial protection measures such as MPAs and IMMAs and to enhance cooperative protection efforts among maritime nations. Moreover, adopting dynamic ocean management approaches, which allow management strategies to shift rapidly in space and time in response to the changing nature of marine environments, can significantly improve conservation outcomes (Maxwell et al. 2015). These dynamic approaches integrate data from multiple sources, including remote sensing, animal tracking, and habitat modeling that incorporates both oceanographic and anthropogenic variables. Once processed and analyzed, the resulting information can be disseminated through real‐time data‐sharing platforms, facilitating timely communication with ocean users and policymakers (Hazen et al. 2018). Furthermore, additional genetic investigations employing high‐resolution molecular approaches, particularly single‐nucleotide polymorphism (SNP) genotyping, whole‐genome sequencing, and complete mitochondrial genome analyses, are warranted for populations distributed along the Indian Ocean coast, as these populations exhibit comparatively greater genetic divergence and appear to be genetically differentiated from their Pacific counterparts. Continued research is therefore warranted to clarify these dynamics, as the Indian Ocean population may be isolated from the rest due to differing oceanographic conditions. Implementing such dynamic management frameworks provides a more adaptive and responsive research and conservation strategy, ensuring that protection measures remain aligned with the shifting distribution, ecological needs, and behavioral patterns of finless porpoises and other vulnerable marine species.

### 4.2 Limitations

While this study provides new insights into the genetic and ecological context of *N. phocaenoides* , certain limitations should be acknowledged. The morphological observations and mitochondrial genome are generated from a single specimen in the Arabian Sea. Although these data provide important baseline insight into genetic variation that is currently underrepresented in existing genomic datasets, broader population‐level sampling will be necessary to more comprehensively assess regional patterns of genetic diversity. The phylogenetic, species delimitation, and haplotypic analyses were conducted using mitochondrial genome data, which effectively capture matrilineal evolutionary patterns. However, incorporating nuclear genomic markers in future studies would provide a more comprehensive understanding of evolutionary relationships and population structure within the genus. In the ecological component, SDM relied on publicly available occurrence records and large‐scale environmental predictors. Such datasets are useful for identifying broad habitat suitability patterns across extensive geographic ranges, although finer‐scale ecological processes may require additional field‐based occurrence records and localized environmental measurements. Thus, the models characterize broad‐scale habitat suitability under mean environmental conditions and may not capture seasonal or interannual oceanographic variability or localized oceanographic processes. Finally, although genomic and ecological analyses were conducted to provide complementary perspectives, more rigorous future studies incorporating both population genetics and spatial ecological data will be necessary to further elucidate evolutionary processes and better inform conservation planning for this species.

## 5 Conclusion

This study presents mitogenomic and ecological analyses of *N. phocaenoides* , addressing critical knowledge gaps particularly in the Arabian Sea. The morphological and craniometric evidence confirmed species identity, while the complete mitochondrial genome exhibited a conserved vertebrate organization under strong purifying selection, indicating functional stability of protein‐coding genes. Further, the phylogenetic reconstruction and haplotype analyses revealed low genetic divergence among *Neophocaena* taxa but identified distinct haplotypic clustering of Arabian Sea lineages. These patterns might suggest potential regional structuring and underscore the significance of the Indian Ocean population as a distinct evolutionary unit. The ensemble model demonstrated high predictive performance and identified that only a limited proportion of the broader range of the species constitutes suitable habitat. Notably, a substantial fraction of these suitable areas lies outside existing protected areas and overlaps with regions subject to intense anthropogenic pressures. Collectively, these findings highlight the vulnerability of *N. phocaenoides* and emphasize the urgent need for expanded spatial protection, strengthened transboundary conservation strategies, and enhanced genomic sampling, particularly in the western portion of its range, to support adaptive management and long‐term conservation planning.

## Author Contributions

**Manokaran Kamalakannan:** conceptualization (equal), data curation (supporting), formal analysis (supporting), funding acquisition (equal), investigation (supporting), methodology (supporting), project administration (supporting), resources (equal), supervision (supporting), writing – original draft (supporting), writing – review and editing (supporting). **Imon Abedin:** data curation (supporting), formal analysis (supporting), methodology (supporting), software (equal), visualization (equal), writing – original draft (supporting). **Angkasa Putra:** data curation (supporting), formal analysis (supporting), methodology (supporting), software (equal), writing – original draft (supporting). **Vishwanath D. Hegde:** data curation (supporting), investigation (supporting), validation (supporting), writing – review and editing (supporting). **Dhriti Banerjee:** funding acquisition (equal), investigation (supporting), project administration (supporting), supervision (supporting), validation (supporting), writing – review and editing (supporting). **Jung Hwa Choi:** investigation (supporting), validation (supporting), writing – review and editing (supporting). **Hyun‐Woo Kim:** investigation (supporting), supervision (supporting), validation (supporting), writing – review and editing (supporting). **Shantanu Kundu:** conceptualization (equal), formal analysis (supporting), methodology (supporting), project administration (supporting), resources (equal), supervision (supporting), visualization (equal), writing – original draft (supporting), writing – review and editing (supporting).

## Funding

This research was supported by the Core Funding of Zoological Survey of India, Kolkata, under the Ministry of Environment, Forest and Climate Change, New Delhi, Government of India and Korea Institute of Marine Science & Technology Promotion (KIMST) funded by the Ministry of Oceans and Fisheries, South Korea (RS‐2026‐25532898).

## Conflicts of Interest

The authors declare no conflicts of interest.

## Supporting information


**Figure S1:** Figure showing the correlations (< 0.8) among covariates selected for the final model for *N. phocaenoides* . The Pearson correlation coefficient was primarily used; however, if the Spearman or Kendall correlation coefficient exceeded the Pearson value, an “s” or “k” is displayed in the bottom‐right corner of the corresponding variable box.
**FIGURE S2:** Schematic representation of the ETAS‐1 region within the CR of *Neophocaena* species.
**FIGURE S3:** Schematic representation of the ETAS‐2 region within the CR of *Neophocaena* species.
**FIGURE S4:** Schematic representation of the CSB domains within the CR of *Neophocaena* species.
**FIGURE S5:** Evaluation plots and variable importance of each algorithm within the ensemble model for *N. phocaenoides* .
**FIGURE S6:** Response curves showing the effects of environmental predictors on the predicted habitat suitability of *N. phocaenoides* across five modeling algorithms (BRT, GLM, MARS, MaxEnt, and RF).
**TABLE S1:** The newly generated (marked with asterisk) and database‐derived mitogenome sequences of *Neophocaena* species/subspecies, along with their accession numbers, were grouped according to their inferred haplotypes.
**TABLE S2:** Mean genetic distance and standard deviation (SD) among *Neophocaena* species/subspecies inferred from 13 mitochondrial PCGs.
**TABLE S3:** Amino acid composition and RSCU values were calculated from the PCGs of *Neophocaena* species/subspecies.
**TABLE S4:** Pairwise Ka/Ks comparisons for each PCGs among *Neophocaena* species/subspecies.
**TABLE S5:** The mitogenome sequences of *Neophocaena* species/subspecies were grouped according to the estimated OTUs determined by two species delimitation methods (ABGD and ASAP).
**TABLE S6:** The habitat (in sq. km.) of *N. phocaenoides* under MPAs and IMMAs within country maritime boundary.
**TABLE S7:** Country wise mean cumulative anthropogenic pressure within the habitat of *N. phocaenoides* within country maritime boundary.

## Data Availability

The assembled mitogenome has been deposited in the GenBank database (https://www.ncbi.nlm.nih.gov/) under accession number OR353710. The ecological modeling data are included in the main text and the Supporting Information. Any additional data related to this study are available from the corresponding authors (Manokaran Kamalakannan and Shantanu Kundu) upon reasonable request.
