# Supplementary material for: Mitogenomic and Ecological Perspectives on the Wide‐Ridged Indo‐Pacific Finless Porpoise Neophocaena phocaenoides: Toward Emerging Conservation Strategies
Source: Ecol Evol. 2026 Sep 18;16(9):e74249. doi: 10.1002/ece3.74249 (PMC13588720; doi:10.1002/ece3.74249)
Supplement: Supplementary file 1 [file ECE3-16-e74249-s001.docx]

**Supporting Information**

**FIGURE S1 |** Figure showing the correlations (< 0.8) among covariates selected for the final model for *N. phocaenoides*. The Pearson correlation coefficient was primarily used; however, if the Spearman or Kendall correlation coefficient exceeded the Pearson value, an ‘s’ or ‘k’ is displayed in the bottom-right corner of the corresponding variable box.

**FIGURE S2 |** Schematic representation of the ETAS-1 region within the CR of *Neophocaena* species.

**FIGURE S3 |** Schematic representation of the ETAS-2 region within the CR of *Neophocaena* species.

**FIGURE S4 |** Schematic representation of the CSB domains within the CR of *Neophocaena* species.

**FIGURE S5 |** Evaluation plots and variable importance of each algorithm within the ensemble model for *N. phocaenoides*.
 **FIGURE S6 |** Response curves showing the effects of environmental predictors on the predicted habitat suitability of *N. phocaenoides* across five modelling algorithms (BRT, GLM, MARS, MaxEnt, and RF).

**TABLE S1 |** The newly generated (marked with asterisk) and database-derived mitogenome sequences of *Neophocaena* species/subspecies, along with their accession numbers, were grouped according to their inferred haplotypes.

| **Sl No.** | **Species** | **Accession No.** | **Haplotype** |
| --- | --- | --- | --- |
| 1 | *N. phocaenoides* | PX427675 | Hap_1 |
| 2 | *N. phocaenoides* | BK071573 | Hap_2 |
| 3 | *N. phocaenoides* | BK071570 | Hap_3 |
| 4 | *N. phocaenoides* | BK071569 | Hap_4 |
| 5 | *N. phocaenoides* | BK071568 | Hap_5 |
| 6 | *N. phocaenoides* | BK071564 | Hap_6 |
| 7 | *N. phocaenoides* | BK071563 | Hap_7 |
| 8 | *N. phocaenoides* | BK071562 | Hap_8 |
| 9 | *N. phocaenoides* | BK071561 | Hap_9 |
| 10 | *N. phocaenoides* | BK071559/BK071557/BK071555 | Hap_10 |
| 11 | *N. phocaenoides* | BK071558 | Hap_11 |
| 12 | *N. phocaenoides* | BK071556/MT948062 | Hap_12 |
| 13 | *N. phocaenoides* | BK071554 | Hap_13 |
| 14 | *N. phocaenoides* | BK071551 | Hap_14 |
| 15 | *N. phocaenoides* | BK071548 | Hap_15 |
| 16 | *N. phocaenoides* | BK071547 | Hap_16 |
| 17 | *N. phocaenoides* | OR353710* | Hap_17 |
| 18 | *N. phocaenoides* | MT948061 | Hap_18 |
| 19 | *N. phocaenoides* | MT948060 | Hap_19 |
| 20 | *N. phocaenoides/N. asiaeorientalis asiaeorientalis* | MT948059/BK071527 | Hap_20 |
| 21 | *N. phocaenoides* | MT948058 | Hap_21 |
| 22 | *N. phocaenoides* | MT948057 | Hap_22 |
| 23 | *N. phocaenoides* | KX650870 | Hap_23 |
| 24 | *N. phocaenoides/N. asiaeorientalis sunameri* | KC777291/BK071543 | Hap_24 |
| 25 | *Neophocaena asiaeorientalis asiaeorientalis* | BK071538 | Hap_25 |
| 26 | *Neophocaena asiaeorientalis asiaeorientalis* | BK071537 | Hap_26 |
| 27 | *Neophocaena asiaeorientalis asiaeorientalis* | BK071536/BK071535/BK071528/BK071526 | Hap_27 |
| 28 | *Neophocaena asiaeorientalis asiaeorientalis* | BK071534/BK071533 | Hap_28 |
| 29 | *Neophocaena asiaeorientalis asiaeorientalis* | BK071532 | Hap_29 |
| 30 | *Neophocaena asiaeorientalis asiaeorientalis* | BK071531/BK071530 | Hap_30 |
| 31 | *Neophocaena asiaeorientalis asiaeorientalis* | BK071529 | Hap_31 |
| 32 | *Neophocaena asiaeorientalis asiaeorientalis* | KU886000 | Hap_32 |
| 33 | *Neophocaena asiaeorientalis asiaeorientalis* | KR108308 | Hap_33 |
| 34 | *Neophocaena asiaeorientalis* | KP170488 | Hap_34 |
| 35 | *Neophocaena asiaeorientalis sunameri* | BK071572 | Hap_35 |
| 36 | *Neophocaena asiaeorientalis sunameri* | BK071571 | Hap_36 |
| 37 | *Neophocaena asiaeorientalis sunameri* | BK071567 | Hap_37 |
| 38 | *Neophocaena asiaeorientalis sunameri* | BK071566 | Hap_38 |
| 39 | *Neophocaena asiaeorientalis sunameri* | BK071565 | Hap_39 |
| 40 | *Neophocaena asiaeorientalis sunameri* | BK071560 | Hap_40 |
| 41 | *Neophocaena asiaeorientalis sunameri* | BK071553/BK071540 | Hap_41 |
| 42 | *Neophocaena asiaeorientalis sunameri* | BK071552/BK071541 | Hap_42 |
| 43 | *Neophocaena asiaeorientalis sunameri* | BK071550 | Hap_43 |
| 44 | *Neophocaena asiaeorientalis sunameri* | BK071549 | Hap_44 |
| 45 | *Neophocaena asiaeorientalis sunameri* | BK071546 | Hap_45 |
| 46 | *Neophocaena asiaeorientalis sunameri* | BK071545 | Hap_46 |
| 47 | *Neophocaena asiaeorientalis sunameri* | BK071544 | Hap_47 |
| 48 | *Neophocaena asiaeorientalis sunameri* | BK071542 | Hap_48 |
| 49 | *Neophocaena asiaeorientalis sunameri* | BK071539 | Hap_49 |
| 50 | *Neophocaena asiaeorientalis sunameri* | PQ560594 | Hap_50 |
| 51 | *Neophocaena asiaeorientalis sunameri* | PQ560593 | Hap_51 |
| 52 | *Neophocaena asiaeorientalis sunameri* | PQ560592 | Hap_52 |
| 53 | *Neophocaena asiaeorientalis sunameri* | PQ560591 | Hap_53 |
| 54 | *Neophocaena asiaeorientalis sunameri* | PQ560590/PQ560574 | Hap_54 |
| 55 | *Neophocaena asiaeorientalis sunameri* | PQ560589 | Hap_55 |
| 56 | *Neophocaena asiaeorientalis sunameri* | PQ560588 | Hap_56 |
| 57 | *Neophocaena asiaeorientalis sunameri* | PQ560587 | Hap_57 |
| 58 | *Neophocaena asiaeorientalis sunameri* | PQ560586 | Hap_58 |
| 59 | *Neophocaena asiaeorientalis sunameri* | PQ560585 | Hap_59 |
| 60 | *Neophocaena asiaeorientalis sunameri* | PQ560584 | Hap_60 |
| 61 | *Neophocaena asiaeorientalis sunameri* | PQ560583 | Hap_61 |
| 62 | *Neophocaena asiaeorientalis sunameri* | PQ560582 | Hap_62 |
| 63 | *Neophocaena asiaeorientalis sunameri* | PQ560581 | Hap_63 |
| 64 | *Neophocaena asiaeorientalis sunameri* | PQ560580 | Hap_64 |
| 65 | *Neophocaena asiaeorientalis sunameri* | PQ560579 | Hap_65 |
| 66 | *Neophocaena asiaeorientalis sunameri* | PQ560578 | Hap_66 |
| 67 | *Neophocaena asiaeorientalis sunameri* | PQ560577 | Hap_67 |
| 68 | *Neophocaena asiaeorientalis sunameri* | PQ560576 | Hap_68 |
| 69 | *Neophocaena asiaeorientalis sunameri* | PQ560575 | Hap_69 |
| 70 | *Neophocaena asiaeorientalis sunameri* | PQ560573 | Hap_70 |
| 71 | *Neophocaena asiaeorientalis sunameri* | PQ560572 | Hap_71 |
| 72 | *Neophocaena asiaeorientalis sunameri* | KX650872 | Hap_72 |
| 73 | *Neophocaena asiaeorientalis sunameri* | KX650871 | Hap_73 |
| 74 | *Neophocaena asiaeorientalis sunameri* | KX650869 | Hap_74 |
| 75 | *Neophocaena asiaeorientalis sunameri* | KT852939 | Hap_75 |
| 76 | *Neophocaena asiaeorientalis sunameri* | KR108307 | Hap_76 |

**TABLE S2 |** Mean genetic distance and standard deviation (SD) among *Neophocaena* species/subspecies inferred from 13 mitochondrial PCGs.

| **Genes** | **Mean** | **SD** |
| --- | --- | --- |
| *ATP6* | 0.004 | 0.003 |
| *ATP8* | 0.008 | 0.007 |
| *COI* | 0.004 | 0.004 |
| *COII* | 0.007 | 0.024 |
| *COIII* | 0.004 | 0.003 |
| *Cytb* | 0.005 | 0.004 |
| *ND1* | 0.004 | 0.003 |
| *ND2* | 0.005 | 0.005 |
| *ND3* | 0.003 | 0.004 |
| *ND4* | 0.004 | 0.005 |
| *ND4L* | 0.007 | 0.008 |
| *ND5* | 0.006 | 0.005 |
| *ND6* | 0.019 | 0.090 |

**TABLE S3 |** Amino acid composition and RSCU values were calculated from the PCGs of *Neophocaena* species/subspecies.

| ***Neophocaena phocaenoides*** | | | | | | | | | | | |
| --- | --- | --- | --- | --- | --- | --- | --- | --- | --- | --- | --- |
| Codon | Count | RSCU | Codon | Count | RSCU | Codon | Count | RSCU | Codon | Count | RSCU |
| UUU(F) | 86 | 0.89 | UCU(S) | 68 | 1.17 | UAU(Y) | 102 | 0.99 | UGU(C) | 21 | 0.98 |
| UUC(F) | 108 | 1.11 | UCC(S) | 80 | 1.38 | UAC(Y) | 105 | 1.01 | UGC(C) | 22 | 1.02 |
| UUA(L) | 97 | 1.15 | UCA(S) | 102 | 1.75 | UAA(*) | 81 | 1.37 | UGA(*) | 63 | 1.06 |
| UUG(L) | 28 | 0.33 | UCG(S) | 23 | 0.40 | UAG(*) | 34 | 0.57 | UGG(W) | 28 | 1.00 |
| CUU(L) | 72 | 0.85 | CCU(P) | 90 | 1.20 | CAU(H) | 54 | 0.71 | CGU(R) | 18 | 0.83 |
| CUC(L) | 97 | 1.15 | CCC(P) | 111 | 1.48 | CAC(H) | 99 | 1.29 | CGC(R) | 23 | 1.06 |
| CUA(L) | 180 | 2.13 | CCA(P) | 84 | 1.12 | CAA(Q) | 95 | 1.56 | CGA(R) | 30 | 1.38 |
| CUG(L) | 34 | 0.40 | CCG(P) | 14 | 0.19 | CAG(Q) | 27 | 0.44 | CGG(R) | 5 | 0.23 |
| AUU(I) | 113 | 0.99 | ACU(T) | 65 | 0.86 | AAU(N) | 86 | 0.84 | AGU(S) | 27 | 0.46 |
| AUC(I) | 115 | 1.00 | ACC(T) | 104 | 1.37 | AAC(N) | 119 | 1.16 | AGC(S) | 49 | 0.84 |
| AUA(I) | 116 | 1.01 | ACA(T) | 116 | 1.53 | AAA(K) | 104 | 1.61 | AGA(R) | 32 | 1.48 |
| AUG(M) | 38 | 1.00 | ACG(T) | 18 | 0.24 | AAG(K) | 25 | 0.39 | AGG(R) | 22 | 1.02 |
| GUU(V) | 23 | 0.84 | GCU(A) | 40 | 0.86 | GAU(D) | 22 | 0.59 | GGU(G) | 30 | 0.90 |
| GUC(V) | 27 | 0.99 | GCC(A) | 75 | 1.60 | GAC(D) | 52 | 1.41 | GGC(G) | 41 | 1.22 |
| GUA(V) | 39 | 1.43 | GCA(A) | 63 | 1.35 | GAA(E) | 47 | 1.34 | GGA(G) | 42 | 1.25 |
| GUG(V) | 20 | 0.73 | GCG(A) | 9 | 0.19 | GAG(E) | 23 | 0.66 | GGG(G) | 21 | 0.63 |
| ***Neophocaena asiaeorientalis asiaeorientalis*** | | | | | | | | | | | |
| Codon | Count | RSCU | Codon | Count | RSCU | Codon | Count | RSCU | Codon | Count | RSCU |
| UUU(F) | 98 | 1.08 | UCU(S) | 71 | 1.18 | UAU(Y) | 107 | 1.08 | UGU(C) | 27 | 0.96 |
| UUC(F) | 83 | 0.92 | UCC(S) | 71 | 1.18 | UAC(Y) | 91 | 0.92 | UGC(C) | 29 | 1.04 |
| UUA(L) | 80 | 0.91 | UCA(S) | 92 | 1.53 | UAA(*) | 53 | 1.09 | UGA(*) | 62 | 1.27 |
| UUG(L) | 26 | 0.29 | UCG(S) | 22 | 0.37 | UAG(*) | 31 | 0.64 | UGG(W) | 37 | 1.00 |
| CUU(L) | 90 | 1.02 | CCU(P) | 101 | 1.44 | CAU(H) | 58 | 0.82 | CGU(R) | 25 | 0.88 |
| CUC(L) | 111 | 1.26 | CCC(P) | 91 | 1.30 | CAC(H) | 84 | 1.18 | CGC(R) | 21 | 0.74 |
| CUA(L) | 183 | 2.08 | CCA(P) | 76 | 1.08 | CAA(Q) | 80 | 1.67 | CGA(R) | 29 | 1.02 |
| CUG(L) | 39 | 0.44 | CCG(P) | 13 | 0.19 | CAG(Q) | 16 | 0.33 | CGG(R) | 18 | 0.64 |
| AUU(I) | 130 | 1.03 | ACU(T) | 82 | 1.03 | AAU(N) | 93 | 0.90 | AGU(S) | 48 | 0.80 |
| AUC(I) | 119 | 0.94 | ACC(T) | 101 | 1.27 | AAC(N) | 113 | 1.10 | AGC(S) | 56 | 0.93 |
| AUA(I) | 130 | 1.03 | ACA(T) | 113 | 1.42 | AAA(K) | 95 | 1.61 | AGA(R) | 41 | 1.45 |
| AUG(M) | 42 | 1.00 | ACG(T) | 22 | 0.28 | AAG(K) | 23 | 0.39 | AGG(R) | 36 | 1.27 |
| GUU(V) | 39 | 1.38 | GCU(A) | 38 | 0.92 | GAU(D) | 21 | 0.70 | GGU(G) | 29 | 0.83 |
| GUC(V) | 22 | 0.78 | GCC(A) | 68 | 1.65 | GAC(D) | 39 | 1.30 | GGC(G) | 39 | 1.12 |
| GUA(V) | 32 | 1.13 | GCA(A) | 54 | 1.31 | GAA(E) | 46 | 1.39 | GGA(G) | 38 | 1.09 |
| GUG(V) | 20 | 0.71 | GCG(A) | 5 | 0.12 | GAG(E) | 20 | 0.61 | GGG(G) | 33 | 0.95 |
| ***Neophocaena asiaeorientalis sunameri*** | | | | | | | | | | | |
| UUU(F) | 99 | 1.08 | UCU(S) | 73 | 1.21 | UAU(Y) | 106 | 1.07 | UGU(C) | 27 | 1.00 |
| UUC(F) | 84 | 0.92 | UCC(S) | 72 | 1.19 | UAC(Y) | 92 | 0.93 | UGC(C) | 27 | 1.00 |
| UUA(L) | 78 | 0.89 | UCA(S) | 91 | 1.50 | UAA(*) | 54 | 1.12 | UGA(*) | 62 | 1.28 |
| UUG(L) | 28 | 0.32 | UCG(S) | 23 | 0.38 | UAG(*) | 29 | 0.60 | UGG(W) | 36 | 1.00 |
| CUU(L) | 89 | 1.02 | CCU(P) | 95 | 1.38 | CAU(H) | 61 | 0.87 | CGU(R) | 26 | 0.90 |
| CUC(L) | 109 | 1.25 | CCC(P) | 93 | 1.35 | CAC(H) | 80 | 1.13 | CGC(R) | 26 | 0.90 |
| CUA(L) | 184 | 2.11 | CCA(P) | 73 | 1.06 | CAA(Q) | 80 | 1.65 | CGA(R) | 27 | 0.93 |
| CUG(L) | 36 | 0.41 | CCG(P) | 14 | 0.20 | CAG(Q) | 17 | 0.35 | CGG(R) | 20 | 0.69 |
| AUU(I) | 132 | 1.04 | ACU(T) | 77 | 0.98 | AAU(N) | 91 | 0.91 | AGU(S) | 47 | 0.78 |
| AUC(I) | 117 | 0.92 | ACC(T) | 104 | 1.32 | AAC(N) | 108 | 1.09 | AGC(S) | 57 | 0.94 |
| AUA(I) | 132 | 1.04 | ACA(T) | 113 | 1.43 | AAA(K) | 94 | 1.61 | AGA(R) | 40 | 1.38 |
| AUG(M) | 39 | 1.00 | ACG(T) | 21 | 0.27 | AAG(K) | 23 | 0.39 | AGG(R) | 35 | 1.21 |
| GUU(V) | 40 | 1.37 | GCU(A) | 42 | 0.98 | GAU(D) | 23 | 0.72 | GGU(G) | 31 | 0.89 |
| GUC(V) | 23 | 0.79 | GCC(A) | 68 | 1.58 | GAC(D) | 41 | 1.28 | GGC(G) | 40 | 1.14 |
| GUA(V) | 31 | 1.06 | GCA(A) | 55 | 1.28 | GAA(E) | 46 | 1.37 | GGA(G) | 38 | 1.09 |
| GUG(V) | 23 | 0.79 | GCG(A) | 7 | 0.16 | GAG(E) | 21 | 0.63 | GGG(G) | 31 | 0.89 |

**TABLE S4 |** Pairwise Ka/Ks comparisons for each PCGs among *Neophocaena* species/subspecies.

| **No** | **ND1** | **ND2** | **COI** | **COII** | **ATP8** | **ATP6** | **COIII** | **ND3** | **ND4L** | **ND4** | **ND5** | **ND6** | **Cytb** |
| --- | --- | --- | --- | --- | --- | --- | --- | --- | --- | --- | --- | --- | --- |
| 1 | 0.134 | 0.096 | 0.030 | 0.038 | 0.203 | 0.108 | 0.035 | 0.323 | 0.118 | 0.100 | 0.057 | 0.046 | 0.052 |
| 2 | 0.134 | 0.128 | 0.029 | 0.038 | 0.203 | 0.071 | 0.035 | 0.323 | 0.118 | 0.100 | 0.057 | 0.046 | 0.070 |
| 3 | 0.121 | 0.086 | 0.032 | 0.038 | 0.203 | 0.071 | 0.000 | 0.323 | 0.118 | 0.100 | 0.067 | 0.046 | 0.052 |
| 4 | 0.169 | 0.103 | 0.023 | 0.033 | 0.201 | 0.108 | 0.000 | 0.326 | 0.068 | 0.117 | 0.106 | 0.046 | 0.000 |
| 5 | 0.121 | 0.091 | 0.032 | 0.038 | 0.203 | 0.071 | 0.000 | 0.323 | 0.118 | 0.117 | 0.057 | 0.046 | 0.052 |
| 6 | 0.189 | 0.109 | 0.032 | 0.038 | 0.203 | 0.108 | 0.000 | 0.323 | 0.118 | 0.083 | 0.057 | 0.046 | 0.052 |
| 7 | 0.134 | 0.077 | 0.012 | 0.025 | 0.000 | 0.130 | 0.000 | 0.320 | 0.136 | 0.095 | 0.148 | 0.137 | 0.019 |
| 8 | 0.134 | 0.077 | 0.012 | 0.025 | 0.000 | 0.130 | 0.000 | 0.320 | 0.136 | 0.095 | 0.131 | 0.092 | 0.039 |
| 9 | 0.149 | 0.086 | 0.030 | 0.038 | 0.203 | 0.071 | 0.035 | 0.323 | 0.118 | 0.100 | 0.055 | 0.046 | 0.052 |
| 10 | 0.121 | 0.091 | 0.032 | 0.038 | 0.203 | 0.071 | 0.000 | 0.323 | 0.118 | 0.100 | 0.057 | 0.046 | 0.052 |
| 11 | 0.149 | 0.086 | 0.030 | 0.038 | 0.203 | 0.071 | 0.035 | 0.323 | 0.118 | 0.100 | 0.055 | 0.046 | 0.052 |
| 12 | 0.134 | 0.091 | 0.030 | 0.038 | 0.203 | 0.071 | 0.070 | 0.323 | 0.118 | 0.100 | 0.057 | 0.046 | 0.052 |
| 13 | 0.149 | 0.086 | 0.030 | 0.038 | 0.203 | 0.071 | 0.035 | 0.323 | 0.118 | 0.100 | 0.055 | 0.046 | 0.052 |
| 14 | 0.134 | 0.091 | 0.030 | 0.038 | 0.203 | 0.108 | 0.070 | 0.323 | 0.118 | 0.100 | 0.055 | 0.046 | 0.052 |
| 15 | 0.189 | 0.109 | 0.032 | 0.038 | 0.203 | 0.108 | 0.000 | 0.323 | 0.118 | 0.079 | 0.057 | 0.046 | 0.052 |
| 16 | 0.134 | 0.109 | 0.029 | 0.038 | 0.203 | 0.071 | 0.035 | 0.323 | 0.104 | 0.100 | 0.057 | 0.046 | 0.050 |
| 17 | 0.121 | 0.091 | 0.032 | 0.033 | 0.203 | 0.071 | 0.000 | 0.323 | 0.000 | 0.095 | 0.057 | 0.046 | 0.047 |
| 18 | 0.000 | 0.342 | 0.000 | 0.311 | 0.000 | 0.000 | 0.000 | 0.330 | 0.118 | 0.000 | 0.625 | 0.000 | 0.000 |
| 19 | 0.134 | 0.091 | 0.030 | 0.038 | 0.203 | 0.071 | 0.070 | 0.323 | 0.118 | 0.100 | 0.057 | 0.046 | 0.052 |
| 20 | 0.149 | 0.091 | 0.030 | 0.033 | 0.203 | 0.071 | 0.035 | 0.323 | 0.118 | 0.095 | 0.055 | 0.046 | 0.052 |
| 21 | 0.121 | 0.091 | 0.032 | 0.038 | 0.203 | 0.071 | 0.000 | 0.323 | 0.090 | 0.100 | 0.067 | 0.046 | 0.052 |
| 22 | 0.192 | 0.069 | 0.017 | 0.038 | 0.304 | 0.140 | 0.000 | 0.323 | 0.090 | 0.111 | 0.077 | 0.000 | 0.034 |
| 23 | 0.192 | 0.069 | 0.017 | 0.038 | 0.203 | 0.140 | 0.000 | 0.323 | 0.136 | 0.111 | 0.077 | 0.000 | 0.034 |
| 24 | 0.134 | 0.077 | 0.012 | 0.025 | 0.000 | 0.130 | 0.000 | 0.320 | 0.116 | 0.095 | 0.136 | 0.092 | 0.020 |
| 25 | 0.126 | 0.077 | 0.030 | 0.043 | 0.309 | 0.064 | 0.040 | 0.325 | 0.116 | 0.119 | 0.102 | 0.000 | 0.037 |
| 26 | 0.244 | 0.065 | 0.017 | 0.033 | 0.203 | 0.140 | 0.032 | 0.323 | 0.090 | 0.106 | 0.077 | 0.000 | 0.032 |
| 27 | 0.243 | 0.069 | 0.017 | 0.038 | 0.304 | 0.140 | 0.000 | 0.323 | 0.090 | 0.111 | 0.077 | 0.000 | 0.034 |
| 28 | 0.192 | 0.069 | 0.017 | 0.038 | 0.203 | 0.140 | 0.000 | 0.323 | 0.090 | 0.111 | 0.088 | 0.000 | 0.034 |
| 29 | 0.149 | 0.069 | 0.017 | 0.038 | 0.203 | 0.140 | 0.000 | 0.323 | 0.090 | 0.111 | 0.077 | 0.000 | 0.034 |
| 30 | 0.149 | 0.069 | 0.017 | 0.038 | 0.203 | 0.140 | 0.000 | 0.323 | 0.090 | 0.111 | 0.077 | 0.000 | 0.034 |
| 31 | 0.192 | 0.069 | 0.017 | 0.038 | 0.203 | 0.140 | 0.000 | 0.323 | 0.090 | 0.111 | 0.077 | 0.000 | 0.034 |
| 32 | 0.192 | 0.069 | 0.017 | 0.038 | 0.203 | 0.140 | 0.000 | 0.323 | 0.090 | 0.111 | 0.077 | 0.000 | 0.034 |
| 33 | 0.192 | 0.086 | 0.017 | 0.038 | 0.304 | 0.140 | 0.000 | 0.323 | 0.090 | 0.111 | 0.077 | 0.000 | 0.034 |
| 34 | 0.192 | 0.069 | 0.017 | 0.038 | 0.203 | 0.140 | 0.000 | 0.323 | 0.090 | 0.111 | 0.077 | 0.000 | 0.034 |
| 35 | 0.192 | 0.069 | 0.017 | 0.038 | 0.203 | 0.140 | 0.000 | 0.323 | 0.090 | 0.111 | 0.077 | 0.000 | 0.034 |
| 36 | 0.149 | 0.069 | 0.017 | 0.038 | 0.203 | 0.140 | 0.000 | 0.323 | 0.090 | 0.111 | 0.077 | 0.000 | 0.052 |
| 37 | 0.149 | 0.069 | 0.017 | 0.038 | 0.203 | 0.140 | 0.000 | 0.323 | 0.090 | 0.111 | 0.077 | 0.000 | 0.034 |
| 38 | 0.192 | 0.069 | 0.017 | 0.038 | 0.304 | 0.140 | 0.000 | 0.323 | 0.090 | 0.111 | 0.077 | 0.000 | 0.034 |
| 39 | 0.149 | 0.069 | 0.017 | 0.038 | 0.203 | 0.140 | 0.000 | 0.323 | 0.090 | 0.111 | 0.077 | 0.000 | 0.034 |
| 40 | 0.149 | 0.069 | 0.017 | 0.091 | 0.203 | 0.140 | 0.000 | 0.323 | 0.090 | 0.111 | 0.074 | 0.000 | 0.034 |
| 41 | 0.126 | 0.065 | 0.017 | 0.038 | 0.310 | 0.081 | 0.000 | 0.323 | 0.078 | 0.095 | 0.077 | 0.000 | 0.045 |
| 42 | 0.149 | 0.069 | 0.072 | 0.044 | 0.203 | 0.140 | 0.000 | 0.323 | 0.090 | 0.111 | 0.077 | 0.000 | 0.034 |
| 43 | 0.126 | 0.077 | 0.029 | 0.043 | 0.309 | 0.122 | 0.040 | 0.325 | 0.078 | 0.100 | 0.102 | 0.000 | 0.037 |
| 44 | 0.192 | 0.069 | 0.017 | 0.038 | 0.203 | 0.140 | 0.000 | 0.323 | 0.090 | 0.106 | 0.077 | 0.000 | 0.052 |
| 45 | 0.126 | 0.073 | 0.029 | 0.043 | 0.309 | 0.139 | 0.040 | 0.325 | 0.078 | 0.106 | 0.102 | 0.000 | 0.037 |
| 46 | 0.126 | 0.073 | 0.027 | 0.043 | 0.309 | 0.092 | 0.040 | 0.323 | 0.078 | 0.106 | 0.102 | 0.000 | 0.037 |
| 47 | 0.126 | 0.073 | 0.029 | 0.043 | 0.309 | 0.092 | 0.040 | 0.407 | 0.078 | 0.106 | 0.102 | 0.000 | 0.037 |
| 48 | 0.126 | 0.073 | 0.029 | 0.043 | 0.309 | 0.092 | 0.040 | 0.325 | 0.078 | 0.106 | 0.099 | 0.000 | 0.037 |
| 49 | 0.126 | 0.058 | 0.029 | 0.043 | 0.309 | 0.092 | 0.040 | 0.325 | 0.078 | 0.095 | 0.119 | 0.000 | 0.056 |
| 50 | 0.126 | 0.077 | 0.029 | 0.043 | 0.309 | 0.122 | 0.040 | 0.325 | 0.078 | 0.095 | 0.102 | 0.000 | 0.037 |
| 51 | 0.192 | 0.069 | 0.017 | 0.038 | 0.203 | 0.122 | 0.000 | 0.323 | 0.078 | 0.101 | 0.077 | 0.000 | 0.049 |
| 52 | 0.192 | 0.069 | 0.018 | 0.077 | 0.203 | 0.140 | 0.000 | 0.323 | 0.090 | 0.106 | 0.077 | 0.000 | 0.034 |
| 53 | 0.244 | 0.069 | 0.017 | 0.038 | 0.203 | 0.122 | 0.000 | 0.323 | 0.090 | 0.097 | 0.077 | 0.000 | 0.049 |
| 54 | 0.126 | 0.096 | 0.029 | 0.038 | 0.309 | 0.092 | 0.079 | 0.325 | 0.078 | 0.095 | 0.119 | 0.000 | 0.037 |
| 55 | 0.189 | 0.109 | 0.032 | 0.038 | 0.203 | 0.071 | 0.000 | 0.323 | 0.118 | 0.083 | 0.055 | 0.046 | 0.052 |
| 56 | 0.244 | 0.065 | 0.017 | 0.033 | 0.203 | 0.140 | 0.032 | 0.323 | 0.090 | 0.106 | 0.077 | 0.000 | 0.032 |
| 57 | 0.126 | 0.077 | 0.029 | 0.043 | 0.309 | 0.122 | 0.040 | 0.325 | 0.078 | 0.095 | 0.102 | 0.000 | 0.034 |
| 58 | 0.126 | 0.077 | 0.029 | 0.043 | 0.309 | 0.122 | 0.040 | 0.325 | 0.078 | 0.095 | 0.102 | 0.000 | 0.037 |
| 59 | 0.126 | 0.058 | 0.029 | 0.043 | 0.309 | 0.092 | 0.040 | 0.325 | 0.078 | 0.095 | 0.119 | 0.000 | 0.056 |
| 60 | 0.126 | 0.069 | 0.013 | 0.030 | 0.309 | 0.071 | 0.040 | 0.325 | 0.078 | 0.101 | 0.090 | 0.000 | 0.075 |
| 61 | 0.112 | 0.073 | 0.029 | 0.038 | 0.203 | 0.140 | 0.040 | 0.325 | 0.078 | 0.106 | 0.107 | 0.000 | 0.034 |
| 62 | 0.126 | 0.077 | 0.026 | 0.033 | 0.309 | 0.081 | 0.032 | 0.323 | 0.136 | 0.132 | 0.086 | 0.000 | 0.034 |
| 63 | 0.126 | 0.077 | 0.029 | 0.088 | 0.309 | 0.092 | 0.040 | 0.325 | 0.078 | 0.095 | 0.107 | 0.000 | 0.037 |
| 64 | 0.112 | 0.069 | 0.029 | 0.043 | 0.309 | 0.140 | 0.040 | 0.325 | 0.078 | 0.095 | 0.107 | 0.000 | 0.052 |
| 65 | 0.112 | 0.069 | 0.029 | 0.043 | 0.309 | 0.140 | 0.040 | 0.325 | 0.078 | 0.095 | 0.107 | 0.000 | 0.034 |
| 66 | 0.112 | 0.073 | 0.029 | 0.038 | 0.203 | 0.081 | 0.040 | 0.325 | 0.078 | 0.106 | 0.107 | 0.000 | 0.034 |
| 67 | 0.112 | 0.073 | 0.027 | 0.043 | 0.309 | 0.081 | 0.000 | 0.325 | 0.078 | 0.090 | 0.102 | 0.000 | 0.034 |
| 68 | 0.112 | 0.073 | 0.029 | 0.043 | 0.309 | 0.081 | 0.079 | 0.325 | 0.078 | 0.095 | 0.107 | 0.000 | 0.034 |
| 69 | 0.144 | 0.069 | 0.017 | 0.038 | 0.310 | 0.064 | 0.000 | 0.387 | 0.078 | 0.090 | 0.059 | 0.000 | 0.063 |
| 70 | 0.112 | 0.069 | 0.027 | 0.038 | 0.309 | 0.081 | 0.040 | 0.325 | 0.078 | 0.090 | 0.107 | 0.000 | 0.052 |
| 71 | 0.168 | 0.077 | 0.042 | 0.030 | 0.309 | 0.071 | 0.040 | 0.323 | 0.136 | 0.117 | 0.104 | 0.000 | 0.056 |
| 72 | 0.112 | 0.073 | 0.027 | 0.043 | 0.309 | 0.081 | 0.000 | 0.325 | 0.078 | 0.095 | 0.102 | 0.000 | 0.034 |
| 73 | 0.112 | 0.073 | 0.029 | 0.038 | 0.309 | 0.081 | 0.040 | 0.325 | 0.078 | 0.095 | 0.102 | 0.000 | 0.032 |
| 74 | 0.144 | 0.069 | 0.017 | 0.038 | 0.310 | 0.108 | 0.000 | 0.387 | 0.078 | 0.090 | 0.059 | 0.000 | 0.047 |
| 75 | 0.126 | 0.077 | 0.029 | 0.088 | 0.309 | 0.081 | 0.040 | 0.325 | 0.078 | 0.095 | 0.107 | 0.000 | 0.037 |
| 76 | 0.112 | 0.073 | 0.029 | 0.043 | 0.309 | 0.092 | 0.079 | 0.325 | 0.078 | 0.095 | 0.107 | 0.000 | 0.059 |
| 77 | 0.126 | 0.077 | 0.029 | 0.088 | 0.309 | 0.092 | 0.040 | 0.325 | 0.078 | 0.095 | 0.107 | 0.000 | 0.034 |
| 78 | 0.126 | 0.077 | 0.029 | 0.088 | 0.309 | 0.092 | 0.040 | 0.325 | 0.078 | 0.095 | 0.102 | 0.000 | 0.034 |
| 79 | 0.112 | 0.073 | 0.027 | 0.043 | 0.309 | 0.092 | 0.040 | 0.325 | 0.078 | 0.095 | 0.099 | 0.000 | 0.034 |
| 80 | 0.112 | 0.073 | 0.027 | 0.038 | 0.309 | 0.092 | 0.040 | 0.325 | 0.078 | 0.095 | 0.099 | 0.000 | 0.052 |
| 81 | 0.112 | 0.069 | 0.029 | 0.043 | 0.309 | 0.140 | 0.040 | 0.325 | 0.078 | 0.095 | 0.107 | 0.000 | 0.034 |
| 82 | 0.112 | 0.073 | 0.029 | 0.043 | 0.309 | 0.092 | 0.040 | 0.325 | 0.078 | 0.095 | 0.119 | 0.000 | 0.034 |
| 83 | 0.100 | 0.073 | 0.029 | 0.043 | 0.309 | 0.092 | 0.035 | 0.325 | 0.078 | 0.095 | 0.107 | 0.000 | 0.034 |
| 84 | 0.134 | 0.103 | 0.014 | 0.038 | 0.203 | 0.071 | 0.035 | 0.323 | 0.118 | 0.090 | 0.059 | 0.046 | 0.047 |
| 85 | 0.189 | 0.109 | 0.017 | 0.038 | 0.203 | 0.071 | 0.000 | 0.323 | 0.118 | 0.083 | 0.065 | 0.046 | 0.052 |
| 86 | 0.121 | 0.096 | 0.015 | 0.038 | 0.203 | 0.108 | 0.035 | 0.323 | 0.118 | 0.100 | 0.057 | 0.046 | 0.052 |
| 87 | 0.168 | 0.086 | 0.018 | 0.033 | 0.227 | 0.188 | 0.000 | 0.323 | 0.090 | 0.106 | 0.077 | 0.000 | 0.034 |
| 88 | 0.149 | 0.069 | 0.017 | 0.038 | 0.203 | 0.140 | 0.000 | 0.323 | 0.090 | 0.111 | 0.088 | 0.000 | 0.034 |
| Average | 0.145 | 0.081 | 0.024 | 0.045 | 0.241 | 0.105 | 0.022 | 0.326 | 0.093 | 0.100 | 0.091 | 0.015 | 0.041 |
| STDEV | 0.038 | 0.031 | 0.009 | 0.032 | 0.074 | 0.031 | 0.023 | 0.013 | 0.022 | 0.014 | 0.062 | 0.027 | 0.012 |

**TABLE S5 |** The mitogenome sequences of *Neophocaena* species/subspecies were grouped according to the estimated OTUs determined by two species delimitation methods (ABGD and ASAP).

| **Operational Taxonomic Units (OTUs)** | **No. of Sequences** | **Accession No.** |
| --- | --- | --- |
| OTU-1 | 2 | PX427675, OR353710 |
| OTU-2 | 82 | BK071573, BK071570, BK071569, BK071564, BK071563, BK071559, BK071558, BK071557, BK071556, BK071555, BK071554, BK071551, BK071548, BK071547, MT948062, MT948061, MT948060, MT948059, MT948058, KX650870, KC777291, BK071538, BK071537, BK071536, BK071535, BK071534, BK071533, BK071532, BK071531, BK071530, BK071529, BK071528, BK071527, BK071526, KU886000, KR108308, BK071572, BK071571, BK071567, BK071566, BK071565, BK071560, BK071553, BK071552, BK071550, BK071549, BK071546, BK071545, BK071544, BK071543, BK071542, BK071541, BK071540, BK071539, PQ560594, PQ560593, PQ560592, PQ560591, PQ560590, PQ560589, PQ560588, PQ560587, PQ560586, PQ560585, PQ560584, PQ560583, PQ560582, PQ560581, PQ560580, PQ560579, PQ560578, PQ560577, PQ560576, PQ560575, PQ560574, PQ560573, PQ560572, KX650872, KX650871, KX650869, KT852939, KR108307 |
| OTU-3 | 1 | BK071568 |
| OTU-4 | 3 | BK071562, BK071561, MT948057 |
| OTU-5 | 1 | KP170488 |

**TABLE S6 |** The habitat (in sq. km.) of *N. phocaenoides* under MPAs and IMMAs within country maritime boundary.

| **Country** | **Area** |
| --- | --- |
| Bahrain | 2069.76 |
| Bangladesh | 10348.8 |
| Cambodia | 846.72 |
| China | 3606.4 |
| India | 11320.96 |
| Indonesia | 9470.72 |
| Iran | 1160.32 |
| Iraq | 752.64 |
| Kuwait | 1693.44 |
| Malaysia | 6397.44 |
| Myanmar | 533.12 |
| Pakistan | 3073.28 |
| Philippines | 972.16 |
| Qatar | 972.16 |
| Saudi Arabia | 2007.04 |
| Sri Lanka | 3669.12 |
| Thailand | 6209.28 |
| United Arab Emirates | 3951.36 |
| Vietnam | 8686.72 |
| Japan | 8498.56 |
| South Korea | 2226.56 |

**TABLE S7 |** Country wise mean cumulative anthropogenic pressure within the habitat of *N. phocaenoides* within country maritime boundary.

| **Country** | **Mean** | **SD** |
| --- | --- | --- |
| Singapore | 1.607 | 0.470 |
| Iraq | 0.868 | 0.242 |
| China | 0.793 | 0.204 |
| Taiwan | 0.752 | 0.242 |
| South Korea | 0.653 | 0.273 |
| Kuwait | 0.636 | 0.310 |
| Qatar | 0.625 | 0.258 |
| Iran | 0.605 | 0.265 |
| Bahrain | 0.582 | 0.289 |
| United Arab Emirates | 0.579 | 0.337 |
| Malaysia | 0.565 | 0.247 |
| Brunei | 0.552 | 0.215 |
| North Korea | 0.518 | 0.182 |
| Pakistan | 0.497 | 0.258 |
| Vietnam | 0.485 | 0.183 |
| Japan | 0.409 | 0.403 |
| Philippines | 0.379 | 0.215 |
| Thailand | 0.377 | 0.281 |
| Saudi Arabia | 0.365 | 0.262 |
| India | 0.357 | 0.293 |
| Sri Lanka | 0.353 | 0.197 |
| Cambodia | 0.352 | 0.201 |
| Oman | 0.315 | 0.156 |
| Indonesia | 0.299 | 0.257 |
| Bangladesh | 0.209 | 0.090 |
| Myanmar | 0.100 | 0.118 |
